# Strategies for Reforestation under Uncertain Future Climates: Guidelines for Alberta, Canada

**DOI:** 10.1371/journal.pone.0022977

**Published:** 2011-08-10

**Authors:** Laura K. Gray, Andreas Hamann

**Affiliations:** Department of Renewable Resources, University of Alberta, Edmonton, Alberta, Canada; University of Washington, United States of America

## Abstract

**Background:**

Commercial forestry programs normally use locally collected seed for reforestation under the assumption that tree populations are optimally adapted to local environments. However, in western Canada this assumption is no longer valid because of climate trends that have occurred over the last several decades. The objective of this study is to show how we can arrive at reforestation recommendations with alternative species and genotypes that are viable under a majority of climate change scenarios.

**Methodology/Principal Findings:**

In a case study for commercially important tree species of Alberta, we use an ecosystem-based bioclimate envelope modeling approach for western North America to project habitat for locally adapted populations of tree species using multi-model climate projections for the 2020s, 2050s and 2080s. We find that genotypes of species that are adapted to drier climatic conditions will be the preferred planting stock over much of the boreal forest that is commercially managed. Interestingly, no alternative species that are currently not present in Alberta can be recommended with any confidence. Finally, we observe large uncertainties in projections of suitable habitat that make reforestation planning beyond the 2050s difficult for most species.

**Conclusion/Significance:**

More than 50,000 hectares of forests are commercially planted every year in Alberta. Choosing alternative planting stock, suitable for expected future climates, could therefore offer an effective climate change adaptation strategy at little additional cost. Habitat projections for locally adapted tree populations under observed climate change conform well to projections for the 2020s, which suggests that it is a safe strategy to change current reforestation practices and adapt to new climatic realities through assisted migration prescriptions.

## Introduction

Reforestation with planting stock that is grown in nurseries is a widely used practice in western Canada and elsewhere. Forest companies and provincial agencies in Alberta plant approximately 80 million seedlings to reforest more than 50,000 hectares annually. For successful reforestation programs, planting stock must be both genetically well adapted to the target environment and contain a sufficient amount of genetic diversity. Generally, two decisions have to be made when selecting planting stock. First, an appropriate species has to be chosen for a planting site. Usually, forest sites can support several tree species, allowing forest managers to choose which species best fit their economic or ecological objectives. The second choice concerns the genetic makeup of reforestation stock. Most widespread tree species show adaptation of local populations to different macroclimatic conditions that are frequently observed over latitudinal or elevational gradients, e.g. [1]. To minimize the risk of maladaptation most jurisdictions legislate seed transfer guidelines or seed zones, which restrict how far seed may be moved from a collection location to a planting site [2], [3]. Under the assumption that local populations are optimally adapted to the environments in which they occur, prescribing reforestation with species and genotypes collected near the planting site can reduce the risk of maladaptation.

**Figure 1:**
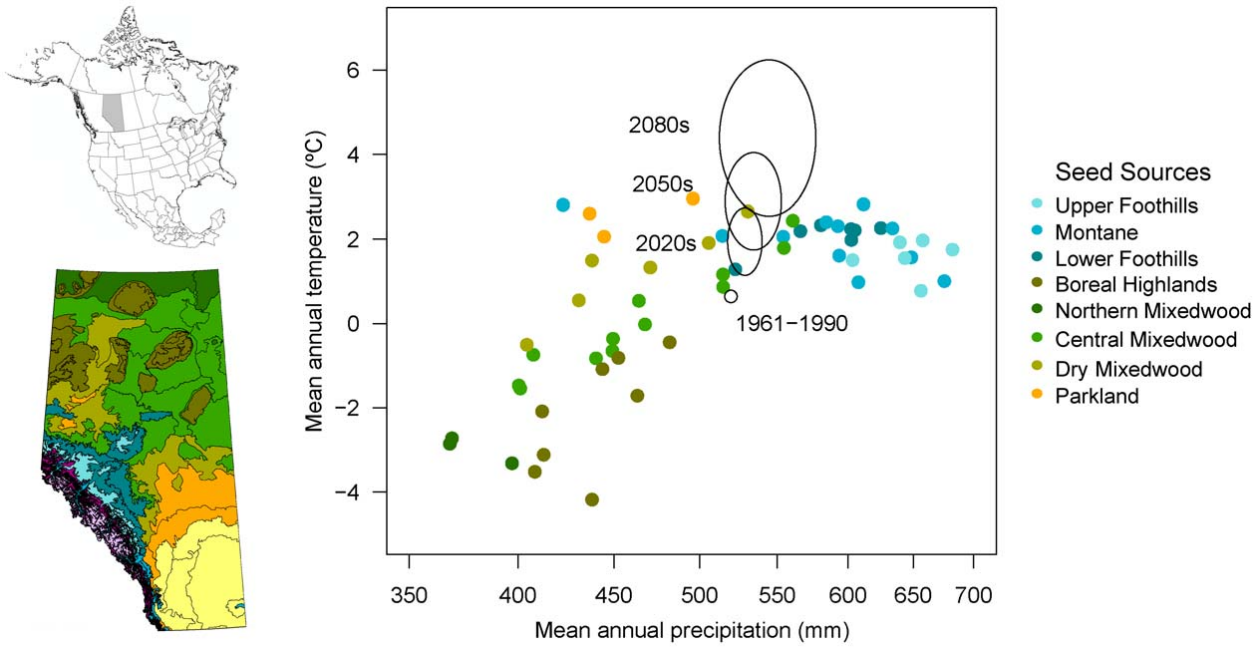
Climate of seed zones in Alberta, which are based on a hierarchical ecological classification system. Colors represent Natural Subregions, and points in the scatterplot represent the finest units of forested ecosystems that govern seed transfer in reforestation. The delineations corresponding to the scatterplot are shown on the map. The expected shift of a mean climate point for Alberta (1961–1990) representing the range of 18 climate change scenarios is indicated by ellipses (2020s, 2050s, 2080s).

In Alberta, movement of seed is regulated with seed zones, a system of approximately 60 geographic delineations for forested areas of the province (Figure 1, map inset). These seed zones are a subdivision of the Alberta Natural Regions and Subregions ecological classification system [4]. Seed can be freely moved within the seed zone or origin, but transferring seed outside seed zone boundaries is usually prohibited [5]. Using fine scale ecosystem classifications as a proxy for the genetic structure of tree species is a common practice when lacking genetic information. As genetic data become available from long-term field experiments, fine scale seed zones are usually consolidated into larger units if no genetic differentiation between adjacent zones is found [2], [3].

Although this system of governing seed movement has been successfully used in many parts of the world, the key assumption that local tree populations are optimally adapted to the environments in which they occur, may no longer be valid. For example, Alberta has experienced a warming trend of 0.8°C and a decrease of about 10% in precipitation over the last 25 years [6]. Reciprocal transplant experiments have shown that there is now a substantial mismatch between local populations and the environments in which they occur, leading to sub-optimal growth [7]. Furthermore, large-scale dieback of forest trees related to drought stress has been observed along the southern edge of the boreal forest [8], [9], [10]. The latter study estimates that drought-related dieback of forest over the last decade has resulted in 45 Megatons of dead biomass in central Alberta, representing 20% of the total aboveground biomass.

Recognizing that management interventions are necessary to maintain forest health and productivity in the face of climate change, the Alberta government released interim seed transfer guidelines in 2009, allowing upward and northward transfers across adjacent seed zone boundaries within the natural subregion of origin [11]. Larger distance seed transfers may be allowed but require case-by-case approval from the Alberta Tree Improvement and Seed Center [11]. We think that this policy framework can be developed into an effective climate change adaptation strategy for the forestry sector, and this study is meant to support decision making by the provincial government of Alberta for selection of species and genotypes that are well adapted to expected future environments.

This study builds on a larger modeling effort that covers 15 commercially important forestry species of western North America [12]. Here, we present a detailed regional analysis that can be used to guide the reforestation activities in Alberta, and that may serve as a template for other jurisdictions. We use multi-model projections of species habitat for the 2020s, 2050s and 2080s to aid species choice for reforestation. The goal is to arrive at species recommendations that are viable under most climate change scenarios. As a second step, we determine suitable genotypes for a given planting site. Given the considerable uncertainty in climate change projections, we provide multiple seed source recommendations that approximately match expected future environments. Multiple seed sources could be prescribed to enhance genetic diversity in the landscape to hedge against uncertainty. We also provide multiple choices of seed sources to allow flexible implementation of assisted migration prescriptions in the face logistical constraints in seed supply that forest companies and provincial agencies face.

## Methods

### Climate envelope modeling

This study builds on an ecosystem-based modeling method developed by Hamann & Wang [13] and Mbogga *et al.* [14]. The approach characterizes the climate space of delineated ecosystem polygons, which represent habitat for individual species populations. The ecosystem units are then predicted as a dependent class variable using climate conditions under various future scenarios as predictor. Predictions were performed with an ensemble classification tree analysis implemented by the RandomForest software package [15] for the R programming environment [16]. RandomForest grows multiple dichotomous decision trees from bootstrap samples to predict a dependent class variable [17]. We used 200 trees in this study, and the final predicted ecosystem was determined by majority vote over all classification trees. As dependent variable, we used the “seedzone” delineation of the Natural Regions and Subregions of Alberta [4]. To determine whether new species or seed sources from outside Alberta should be introduced under climate change scenarios, we expanded the model coverage to Canada and the United States west of 100° longitude. Additional ecosystem units include the “variant” level of the Biogeoclimatic Ecological Classification system for British Columbia [18]. For other Canadian provinces we used the “ecodistrict” level of the National Ecological Framework for Canada [19], and for the United States we used the “level IV” classification of the Ecoregion System [20]. From each of these ecosystem classes we randomly sampled 100 grid cells at 1 km resolution, which we climatically characterized, and subsequently used as training data for classification tree analysis.

### Climate data and climate projections

We used interpolated climate data for the 1961–1990 normal period, covering the United States and Canada west of 100° longitude. Interpolation of weather station data was performed with the Parameter Regression of Independent Slopes Model [21] for monthly minimum temperature, maximum temperature and precipitation. We enhanced this data with lapse-rate based down-scaling to 1 km resolution and an estimation of biologically relevant climate variables with a software package that is freely available at http://www.ualberta.ca/~ahamann/climate.html [6], [22]. Ten predictor variables with low collinearity were chosen, representing both seasonal and annual climate variables. This includes mean annual temperature, mean warmest month temperature, mean coldest month temperature, continentality (difference between mean January and mean July temperature), mean annual precipitation, growing season precipitation (May to September), the number of frost free days, and the number of growing degree days above 5°C. These variables are described in more detail by Wang *et al.* [23]. We also included two dryness indices: annual and summer climate-moisture index according to Hogg [24].

To generate future climate projections for the 2020s, 2050s and 2080s we overlaid projections from general circulation models expressed as the difference from the 1961–1990 normal using the same software package as above. For each future period, climate projections were based on four SRES emission and population growth scenario families (A1FI, A2, B1, B2), implemented by five modeling groups (CGCM, Canada; CSIRO2. Australia; HADCM3, United Kingdom; ECHAM4, Europe; and PCM, United States). Two model-emission scenario combinations (ECHAM4-A1FI and ECHAM4-B1) were unavailable, resulting in 18 climate projections per time period. Similar to GCM projections, recent climate conditions can be expressed as difference from the 1961–1990 normal period (also referred to as anomaly). We use the 1997–2006 decadal anomaly to represent observed climate change over a 25-year period (the midpoint of the 1961–1990 climate baseline period and the midpoint of the recent decadal average: 1975 to 2000).

### Species projections and model validation

We use projected ecosystem units to represent populations of tree species and to derive predictions of species habitat. The frequency and probability of presence of major forest tree species in ecosystem units was calculated from 54,716 forest inventory plots covering western North America. This includes provincial databases from British Columbia previously described in Hamann *et al.* [25]. For Alberta we used permanent and temporary forest inventory plots as well as the Ecological Site Information System (ESIS) database provided by the Government of Alberta [26]. For all the sample plots in western Canada, an estimated percent areal cover of the canopy projected to the ground, scaled by the total canopy of the forest inventory plot was used for species frequency. In the western United States we rely on the Forest Inventory and Analysis database [27], where we used the percent basal area was used as a proxy for frequency because the percent areal cover of the canopy was unavailable. Species frequency for each ecosystem unit was calculated as the average across all sample plots that fall within an ecosystem polygon. The probability of presence of a species was simply calculated as the proportion of the inventory plots within the ecosystem polygon where the species was present.

To assess the predictive accuracy of bioclimate envelope models for individual species, we calculate the area under the curve (AUC) of the receiver operating characteristics (ROC) curve of the probability of species presence. The AUC value measures the ability of the model to detect a species where it is known to be present against its ability to correctly predict where the species is known to be absent [28], [29]. All ROC and AUC calculations were carried out with the ROCR package [30] for the R programming environment [16].

Five commercially important conifer tree species occur in Alberta: black spruce (*Picea mariana* (Miller) Britton), Douglas-fir (*Pseudotsuga menziesii* (Mirbel) Franco), lodgepole pine (*Pinus contorta* Douglas ex Loudon), jack pine (*Pinus banksiana* Lambert, Descr.), and white spruce (*Picea glauca* (Moench) Voss). Ponderosa pine (*Pinus ponderosa* Douglas ex Lawson & C. Lawson) is projected to gain suitable habitat in Alberta in the future [12] and was therefore also included in this analysis. The scientific names are according to the Flora of North America Editorial Committee [31].

### Seed source recommendations

Multiple options of seed sources for reforestation under current and future climates were derived with a multivariate measure of climate similarity. The objective was to find seed sources that best match a target region under observed and projected climate change. To quantify this match, we use the squared Mahalanobis distance, calculated with the Ecodist package [32] for the R programming environment [16]. Mahalanobis distances matrices were calculated for 10 climate predictor variables described above, and are reported for seed zone units characterized under current climate and under ensemble scenarios for the 2020s, 2050s, and 2080s. The Mahalanobis distance is a normalized Euclidean distance that weighs individual variables according to their collinearity with all other variables [33]. Variables that are perfectly correlated are weighted as a single variable in distance calculations, while the Mahalanobis distance for completely independent variables would equal the Euclidean distance. We transformed all climate variables individually to conform to a normal distribution before distance calculations. The Ecodist package further transforms all variables into units of standard deviations around a variable mean of zero prior distance calculations, so that the weight of climate variables is independent of their units of measurement.

## Results

### Alberta climatology and climate change projections

The climatology of Alberta's ecological regions and seed zones is primarily driven by a latitudinal temperature gradient, and precipitation patterns that are related to the regional topography. The Rocky Mountain Foothill and Montane ecosystems receive the largest amounts of precipitation (500–700 mm) with mean annual temperatures around 2°C (Fig. 1, blue shades). Note that the outlying Montane ecosystem represents the Cypress Hill region, a forest island in the southeast of the province's grasslands (yellow). Parklands (orange) represent a transitional zone between grasslands and the boreal forest. Ecosystems of the boreal forest (Fig. 1, green shades) span a diagonal from approximately 400 mm precipitation and −4°C temperature to 500 mm precipitation and 2°C temperature. The diagonal arrangement of Natural Subregion classes (shades of green) suggests that the precipitation/evaporation balance distinguishes these major ecosystem classes.

To visualize projected climate change relative to the 1961–1990 normal climatology, we added the current climatology and projections for a central boreal forest location, an area centered around 56° latitude and 115° longitude (Fig. 1, open circle). The range of uncertainty in predicted temperature and precipitation values is represented by ellipses. The range of projected climate change varies for different locations in Alberta and cannot be comprehensively visualized in this plot. It is clear, however, that the uncertainty in climate change projections stands in strong contrast to the precision with which reforestation is managed trough seed zones at present (each point in Fig. 1 represents a separate seed zone). Even for the 2020s, similar ellipses drawn at other locations may easily encompass several seed zones as possible alternatives for obtaining reforestation material under climate change. At least in this simple, two-dimensional visualization, it appears challenging to pinpoint seed zone recommendations for the 2050s and 2080s, where similar ellipses drawn at various locations may regularly span several ecological subregions, indicated by different colors in Fig. 1.

### Projections of tree species habitat

Area Under the Curve (AUC) statistics suggest that the predictive accuracy of the ecosystem-based climate envelope model for Alberta is satisfactory (Table 1). Local AUC statistics for Alberta are similar to those for the global species range predictions. In general terms, AUC values above 0.9 indicate excellent predictive accuracy and AUC values above 0.8 indicate good accuracy. An AUC value of 0.8 means that 80% of the time a random sample from presence predictions will have a score greater than a random selection from absence predictions across all available probability thresholds to define a presence prediction. An AUC value of 0.5 therefore indicates a random predictor and values between 0.5 and 0.6 are generally considered a failed model [29].

**Table 1:** Species statistics and model accuracy.

	Global Statistics			Alberta Statistics		
Species	Presence Samples^1^	Range Size (square km)	AUC	Presence Samples^2^	Range Size (square km)	AUC
Black spruce	4,489	710,748	0.90	1,750	385,708	0.85
White spruce	7,115	848,866	0.88	3,606	438,013	0.79
Douglas-fir	8,808	1,002,592	0.88	269	9,952	0.91
Lodgepole pine	11,275	1,016,718	0.82	3,813	219,364	0.79
Ponderosa pine	3,967	591,394	0.88	0	0	NA
Jack pine	325	229,194	0.99	322	201,255	0.97

1 Out of 54,716 sample plots, including non-forested plots.

2 Out of 16,391 sample plots, including non-forested plots.

**Figure 2:**
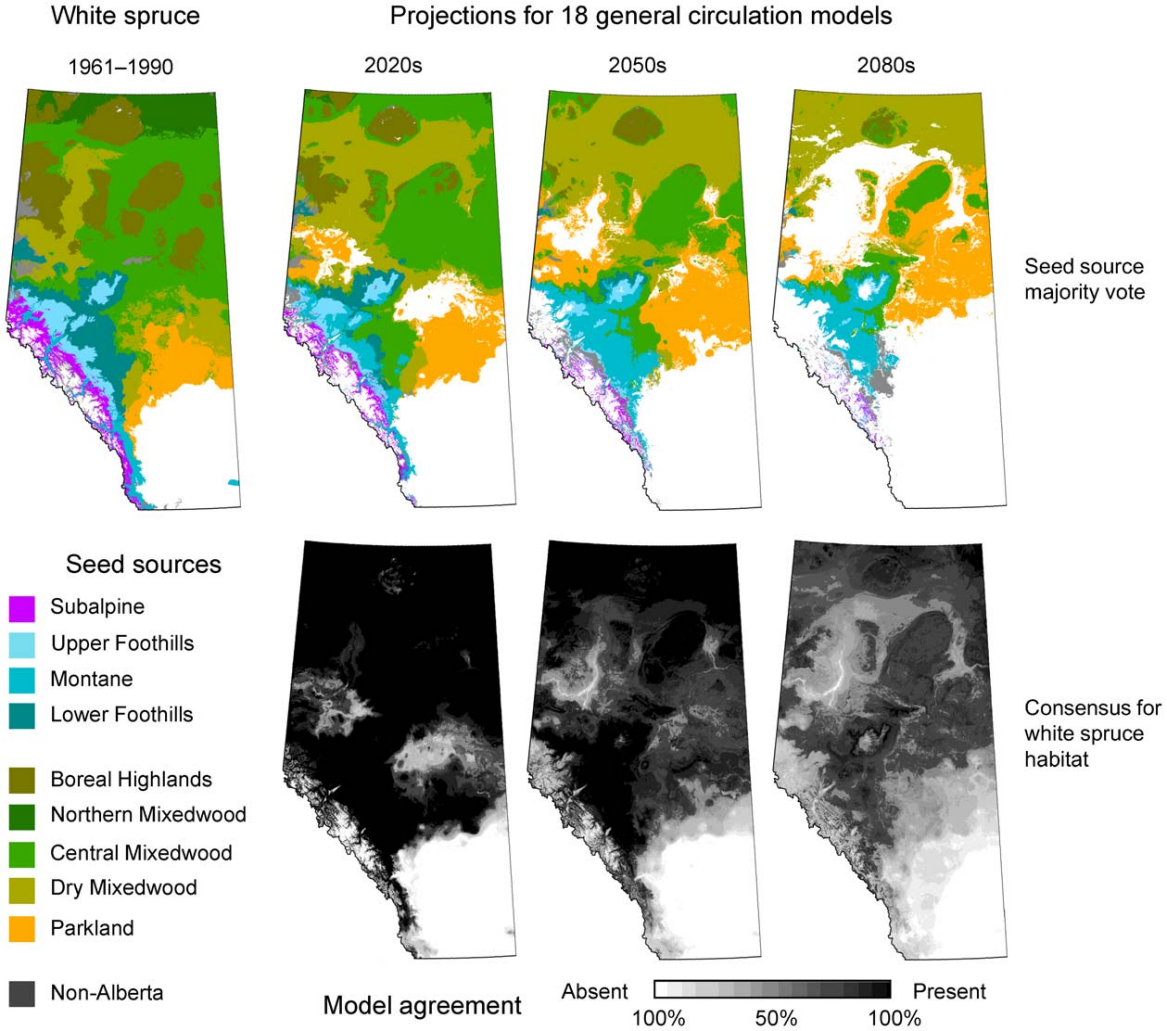
Seed zones projections and consensus of habitat maintenance under projected climate change for white spruce in Alberta. Colors represent broad seed sources corresponding to Natural Subregions (upper row), and the gray scale represents the consensus that habitat is maintained for white spruce for 18 climate change scenarios for the 2020s, 2050s, 2080s (lower row). We require at least a 70% probability that habitat is maintained to make a seed source recommendation.

Habitat projections under future climate change scenarios are shown in Fig. 2 for white spruce. Projections for other important forestry species in Alberta are provided as Fig. S1 (black spruce), Fig. S2 (Douglas-fir), Fig. S3 (lodgepole pine), Fig. S4 (jack pine). In these figures, the black-and-white maps represent the consensus of projections for 18 climate change scenarios. Black indicates that all models agree that climate conditions will be suitable for a species, and white indicates that all models agree that suitable habitat is not available under any scenario. Grey shades represent varying levels of uncertainty in future habitat availability. The results for white spruce are numerically summarized in Table 2, where habitat suitability is provided for selected seed zones of Alberta (complete tables for all species are provided as Tables S1, S2, S3, S4, S5).

**Table 2:** Suitable white spruce habitat expressed as % area of seed zone for observed climate, and expressed as probability of habitat maintenance under climate change projections from 18 general circulation models.

White spruce	Observed climate		Projected climate		
seed zones^1^	1961–1990	1997–2006	2020s	2050s	2080s
CM 1.1	100%	100%	100%	98%	75%
CM 1.2	100%	100%	100%	92%	67%
CM 1.3	100%	100%	100%	98%	71%
DM 1.1	100%	100%	99%	85%	56%
DM 1.2	99%	98%	88%	66%	50%
DM 1.3	100%	100%	74%	74%	59%
DM 2.1	73%	95%	74%	88%	57%
DM 2.2	99%	99%	67%	87%	69%

1 A complete table for all white spruce seed zones is provided as Table S5.

For white spruce (Fig. 2, Table 2), habitat is generally well maintained into the future except for some of the current Dry Mixedwood and transitional Parkland ecosystems. The ecosystem-based habitat projections also convey where appropriate seed sources for expected future climates may be found. For white spruce we observe that seed sources adapted to drier and warmer conditions (Parkland, Dry Mixedwood) should be suitable for an increasing land base in Alberta in the future. In contrast, black spruce is predicted to lose much of its climatically suitable habitat in Alberta, especially in low elevation regions (Fig. S1, Table S1). Douglas-fir is only a commercially viable forestry species in Montane ecosystems in the southeast corner of the province. However, habitat projections for Douglas-fir come with large uncertainties (Fig. S2, Table S2). Climate scenarios that project substantially increased temperature and precipitation for southwestern Alberta, such as the CGCM-A1F1 scenario, result in largely extended habitat for Douglas-fir throughout the Foothill ecosystems of Alberta. On average, however, suitable habitat remains constant or is slightly reduced. The current distribution of lodgepole pine in the foothills of Alberta appears to be well maintained with reasonable certainty (Fig. S3, Table S3). Lastly, habitat for jack pine, currently concentrated at lower elevations in the northeast of the province, is predicted to rapidly decline under most climate change scenarios (Fig. S4, Table S4).

**Figure 3:**
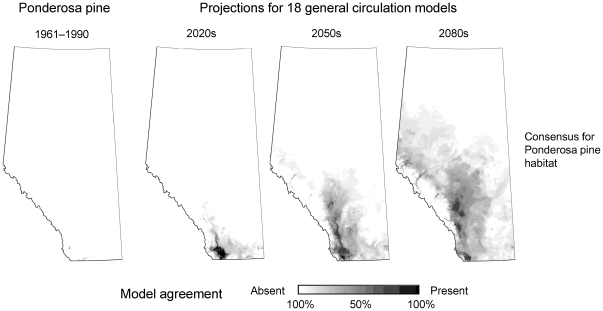
Suitable habitat under projected under climate change for ponderosa pine in Alberta. There is large uncertainty whether this species may become a viable forestry species in Alberta, with extensive areas of suitable habitat projected under some climate change scenarios, and virtually no habitat under other climate change projection.

Notably, no alternative species that are currently not present in Alberta can be recommended with confidence, meaning that suitable habitat is predicted under a clear majority of climate change scenarios. Ponderosa pine (Fig. 3, Table 3) comes closest in gaining habitat with sufficient confidence across multiple climate change scenarios. By the 2050s, the most southern Montane ecosystems of Alberta may become suitable according to approximately half the 18 climate change scenarios we used.

**Table 3:** Suitable ponderosa pine habitat expressed as % area of seed zone for observed climate, and expressed as probability of habitat maintenance under climate change projections from 18 general circulation models.

Ponderosa pine	Observed Climate		Projected Climate		
Seed zones	1961–1990	1997–2006	2020s	2050s	2080s
CM 3.5	0%	0%	0%	11%	43%
DM 2.3	0%	3%	0%	40%	66%
LF 2.3	0%	3%	1%	42%	67%
M 1.1	0%	0%	7%	33%	30%
M 2.1	0%	0%	0%	13%	58%
M 2.2	0%	1%	2%	19%	44%
M 3.2	0%	1%	0%	11%	36%
M 4.3	0%	0%	4%	30%	46%
M 4.4	0%	0%	4%	49%	67%
M 4.5	0%	25%	19%	54%	51%
M 5.6	0%	1%	10%	46%	60%

### Projections of appropriate seed sources

If habitat for a species is maintained under at least 70% of the climate change scenarios, we also provide projections of suitable seed sources. These projections are visualized in the series of color maps in Fig. 2 and Figures S1, S2, S3, S4. In these figures, the colors represent the broad Natural Subregions rather than individual seed zones for the purpose of better visualizing shifts in climate habitat. For white spruce, it is apparent that much of the land base of Alberta will require reforestation stock that is adapted to the warmer and drier ecosystems of the current Dry Mixedwood and Parkland ecosystems. In Table 4, more detailed information is provided for individual seed zones. This table provides alternative seed sources according to the climate match under current and expected future climates. For example, by the 2020s the Central Mixedwood seedzone CM 1.1 is predicted to closely match current Dry Mixedwood climate of the seed zone DM 1.1, or the more southern Central Mixedwood seed zones CM 1.2 and CM 1.3. These seed zones are also close matches under observed climate change, represented by the 1997–2006 average climate, and might therefore be recommended as source for planting material under a climate change adaptation strategy. Complete tables for all seed zones and up to 10 alternative choices are provided as Tables S6, S7, S8, S9, S10. Locations of recommended seed choices originating outside of Alberta are provided as Table S11.

**Table 4:** Seed zones with the best climate match, as measured with the multivariate Mahalanobis distances given in parenthesis.

	Observed Climate		Projected Climate		
Seedzones^1^	1961–1990	1997–2006	2020s	2050s	2080s
CM 1.1	CM11(0), PAD11(0), AP11(0.1), CM13(0.6)	CM12(3.6), DM11(4), CM21(4.8)	DM11(1.6), CM12(2.1), CM13(2.1), PAD11(2.2)	DM11(3.9), CM31(4.8)	[MT]42i(5.2)
CM 1.2	CM12(0), CM21(0.4), DM11(0.4), CM22(1.2)	CM12(3.2), CM24(3.2), CM21(3.6), CM23(3.6)	DM11(1.5), CM12(2), CM21(2.3), CM31(2.4)	CM31(3), DM21(3.3), DM12(4.1), CM32(4.3)	[MT]42i(3.8), 42k(4.2), DM21(4.3), CM32(5.2)
DM 1.2	DM12(0), DM13(0.8), LBH16(0.8), PRP11(0.8)	DM12(2.3), PRP11(2.6), DM13(2.6), CM31(3.6)	PRP11(0.4), DM13(0.6), DM12(1), DM21(1.3)	PRP11(1.2), DM21(1.4), CP11(1.5), DM13(1.5)	CP11(1.7), CP12(1.9), NF11(2), DM22(2.3)
DM 1.3	DM13(0), PRP11(0.3), CM33(0.6), DM12(0.8)	PRP11(2), DM13(2), NF11(2.3), MG11(2.3)	DM13(0.6), CM34(0.9), PRP11(0.9), DM21(1)	CP11(1.3), CP12(1.5), DM22(1.5), CM34(1.6)	DM22(2.2), CP11(2.3), CP12(2.7), CM34(2.9)

Recommendations for U.S. seed sources are preceded by their state of origin.

1 Complete tables for all seed zones with up to 10 alternative options is provided in Tables S6, S7, S8, S9, S10.

## Discussion

### Species choice for reforestation

To minimize the probability of plantation failure in the face of uncertain future climates, we think that the best strategy is to ensure that species habitat is maintained under a wide range of potential climate change scenarios. In this study we restrict our reporting to a threshold of at least 70% of the models to agreeing that species habitat will be maintained. Practitioners may want to set higher thresholds for implementing large-scale reforestation programs to minimize risks of plantation failure. On the other hand, it should be noted that predicted loss of habitat does not necessarily mean dieback or failure to reproduce for tree species. Like most species distribution models, our approach predicts the realized niche (that is the climate space where the species is found to occur naturally) and not the larger fundamental niche space (namely, all climate conditions that a species can tolerate).

By their nature, the predictions of the realized niche space are more conservative as they account for biotic interactions. For example, a tree species may be predicted to lose habitat because it will be out-competed by other species that are better adapted to the predicted environment. However, in a planting environment with site preparation, controlled spacing, and removal of competing vegetation, natural competition would be limited. Secondly, the realized niche of trees may be determined by the ability of seedlings to germinate under favorable conditions and saplings to get established. Mature trees that have access to water through a large root system tend to have a much larger fundamental niche space than their offspring. Again, forest managers can literally “push the envelope” of where a tree species can be successfully grown by cultural treatments, such as planting sturdy seedlings that were grown to a relatively large size in a forest nursery.

Biotic interactions that are implicitly included in realized niche models also include insect pests and diseases. A tree species might be excluded from an area not because the environmental conditions are unfavorable, but because the abiotic conditions are also favorable for a forest pest to which the species is susceptible. This mechanism might be particularly relevant to this study area, as many insects and diseases are excluded from boreal environments due to extreme cold in winter [34]. Species choice in large-scale reforestation programs should be determined by the maintenance of the realized niche under most climate change scenarios, avoiding potential exposure of forest trees to pests and diseases under a continued warming trend (Tables S1, S2, S3, S4, S5 describe where the realized niche space is maintained).

### Choice of genotypes for reforestation

Matching genotypes to abiotic environments with the precision of Alberta's current system of seed zones is unlikely to be a sensible strategy in the face of uncertain future climates. In fact, the current level of precision may not even be necessary under constant climate conditions. Forest trees are normally adapted to broad environmental gradients with substantial within-population genetic diversity [35]. Recent data from genetic provenance experiments suggests that genetic differentiation of tree populations in Alberta would occur at a much broader scale than the current seed zone delineations [3], [36], [37]. As such data from long-term trials become available for more species, general seed zones could be consolidated into larger units to ease the administrative and logistical burden of maintaining many separate seed collections for reforestation needs. For this decision process, which should synthesize genetic differentiation of tree populations, topo-edaphic characteristics of seed zones, and climatic information, we contribute a matrix of climatic similarity for current seed zones in Table S6.

For the development of reforestation strategies under climate change, we encourage practitioners to consult Tables S6 and S7, which provide multiple choices of appropriate seed sources for climate conditions observed over a recent decade and projections for the 2020s. Ideally, seed sources should be used that appear as options under the 1961–1990 reference climate, under 1997–2006 climate, and under 2020s climate projections. Several, consistently suitable choices can usually be found. Making recommendations for the 2050s and 2080s becomes difficult because of the large uncertainties associated with climate projections in the more distant future. We propose that this information might be used for long-term planning, but not for guidance of seed sources in the near future. Planting trees for 2050s and 2080s climate is not sensible as seedlings will likely not survive current planting environments. Also, we ultimately do not need to adapt to a “median climate change scenario” but to climate trends that eventually materialize in Alberta. At this point, we do not know with any reasonable amount of certainty what those conditions will be by the end of the century.

In choosing seed sources for the immediate future, we should further discuss the meaning of the Mahalanobis distances provided in Tables S6, S7, S8, S9, S10. The values provide a measure of climatic similarity (smaller = more similar) between seed zones under 1961–1990 reference climate and future climate conditions expected for these seed zones. The measure does not have an interpretable dimension, and a larger distance does not necessarily imply maladaptation of tree populations. Although this could be the case, it should be noted that we do not have biological and genetic data that demonstrates reduced fitness or productivity as a function of any particular climate variable that is used for the Mahalanobis distance calculation. Nevertheless, the alternate choices provided in Tables S6, S7, S8, S9, S10 could still be used to develop a simple portfolio strategy of adaptation to climate change, where multiple seed sources that approximately match current and 2020s climate are prescribed for reforestation. Such a portfolio approach could continue to use the current seed zone delineations as target areas. Use of multiple seed sources should also include a mechanism for tracking reforestation success, growth, and forest health of plantations to allow recursive improvements [38].

Finally, we should note that importing seed and species from other jurisdictions does not promise to be an important element of a climate change adaptation strategy for the forestry sector in Alberta. Only in small areas of the southern Rocky Mountain Montane and Foothill ecosystem, habitat is projected to be suited to populations originating in montane ecosystems of British Columbia, and the dry conifer forests in Montana, South Dakota, and Wyoming (Table S11). Of approximately 50 western North American tree species that we investigated in a larger modeling effort, no alternative species that are currently not present in Alberta can be recommended with any confidence for reforestation under projected climate change.

## Supporting Information

Figure S1 **Seed zones projections and consensus of habitat maintenance under projected climate change for black spruce in Alberta.** Colors represent broad seed sources corresponding to Natural Subregions (upper row), and the gray scale represents the consensus that habitat is maintained for black spruce under 18 climate change scenarios for the 2020s, 2050s, 2080s (lower row). We require at least a 70% probability that habitat is maintained to make a seed source recommendation.
(PDF)

Figure S2 **Seed zones projections and consensus of habitat maintenance under projected climate change for Douglas-fir in Alberta.** Colors represent broad seed sources corresponding to Natural Subregions (upper row), and the gray scale represents the consensus that habitat is maintained for Douglas-fir under 18 climate change scenarios for the 2020s, 2050s, 2080s (lower row). We require at least a 70% probability that habitat is maintained to make a seed source recommendation.
(PDF)

Figure S3 **Seed zones projections and consensus of habitat maintenance under projected climate change for logepole pine in Alberta.** Colors represent broad seed sources corresponding to Natural Subregions (upper row), and the gray scale represents the consensus that habitat is maintained for lofgepole under 18 climate change scenarios for the 2020s, 2050s, 2080s (lower row). We require at least a 70% probability that habitat is maintained to make a seed source recommendation.
(PDF)

Figure S4 **Seed zones projections and consensus of habitat maintenance under projected climate change for jack pine in Alberta.** Colors represent broad seed sources corresponding to Natural Subregions (upper row), and the gray scale represents the consensus that habitat is maintained for jack pine under 18 climate change scenarios for the 2020s, 2050s, 2080s (lower row). We require at least a 70% probability that habitat is maintained to make a seed source recommendation.
(PDF)

Table S1 **Suitable black spruce habitat expressed as % area of seed zone for observed climate, and expressed as probability of habitat maintenance under climate change projections from 18 general circulation models.**
(PDF)

Table S2 **Suitable Douglas-fir habitat expressed as % area of seed zone for observed climate, and expressed as probability of habitat maintenance under climate change projections from 18 general circulation models.**
(PDF)

Table S3 **Suitable lodgepole pine habitat expressed as % area of seed zone for observed climate, and expressed as probability of habitat maintenance under climate change projections from 18 general circulation models.**
(PDF)

Table S4 **Suitable jack pine habitat expressed as % area of seed zone for observed climate, and expressed as probability of habitat maintenance under climate change projections from 18 general circulation models.**
(PDF)

Table S5 **Suitable white spruce habitat expressed as % area of seed zone for observed climate, and expressed as probability of habitat maintenance under climate change projections from 18 general circulation models.**
(PDF)

Table S6 **Table of best matching seed sources for 1961–1990 climate.** The multivariate Mahalanobis climate distance is given in parenthesis.
(PDF)

Table S7 **Table of best matching seed sources for 1997–2006 climate.** The multivariate Mahalanobis climate distance is given in parenthesis.
(PDF)

Table S8 **Table of best matching seed sources for 2020s climate.** The multivariate Mahalanobis climate distance is given in parenthesis.
(PDF)

Table S9 **Table of best matching seed sources for 2050s climate.** The multivariate Mahalanobis climate distance is given in parenthesis.
(PDF)

Table S10 **Table of best matching seed sources for 2080s climate.** The multivariate Mahalanobis climate distance is given in parenthesis.
(PDF)

Table S11 **Locations of recommended seed choices which originate outside of Alberta.** For British Columbia we report the relevant ecological “variants” and “zones” [18], and for the United States we report the corresponding state and “level III & IV” ecoregions [20].
(PDF)
