# Supplementary material for: Strategies for Reforestation under Uncertain Future Climates: Guidelines for Alberta, Canada
Source: PLoS One. 2011 Aug 10;6(8):e22977. doi: 10.1371/journal.pone.0022977 (PMC3154268; doi:10.1371/journal.pone.0022977)
Supplement: Figure S2 — Seed zones projections and consensus of habitat maintenance under projected climate change for Douglas-fir in Alberta. Colors represent broad seed sources corresponding to Natural Subregions (upper row), and the gray scale represents the consensus that habitat is maintained for Douglas-fir under 18 climate change scenarios for the 2020s, 2050s, 2080s (lower row). We require at least a 70% probability that habitat is maintained to make a seed source recommendation. (PDF) [file pone.0022977.s002.pdf]

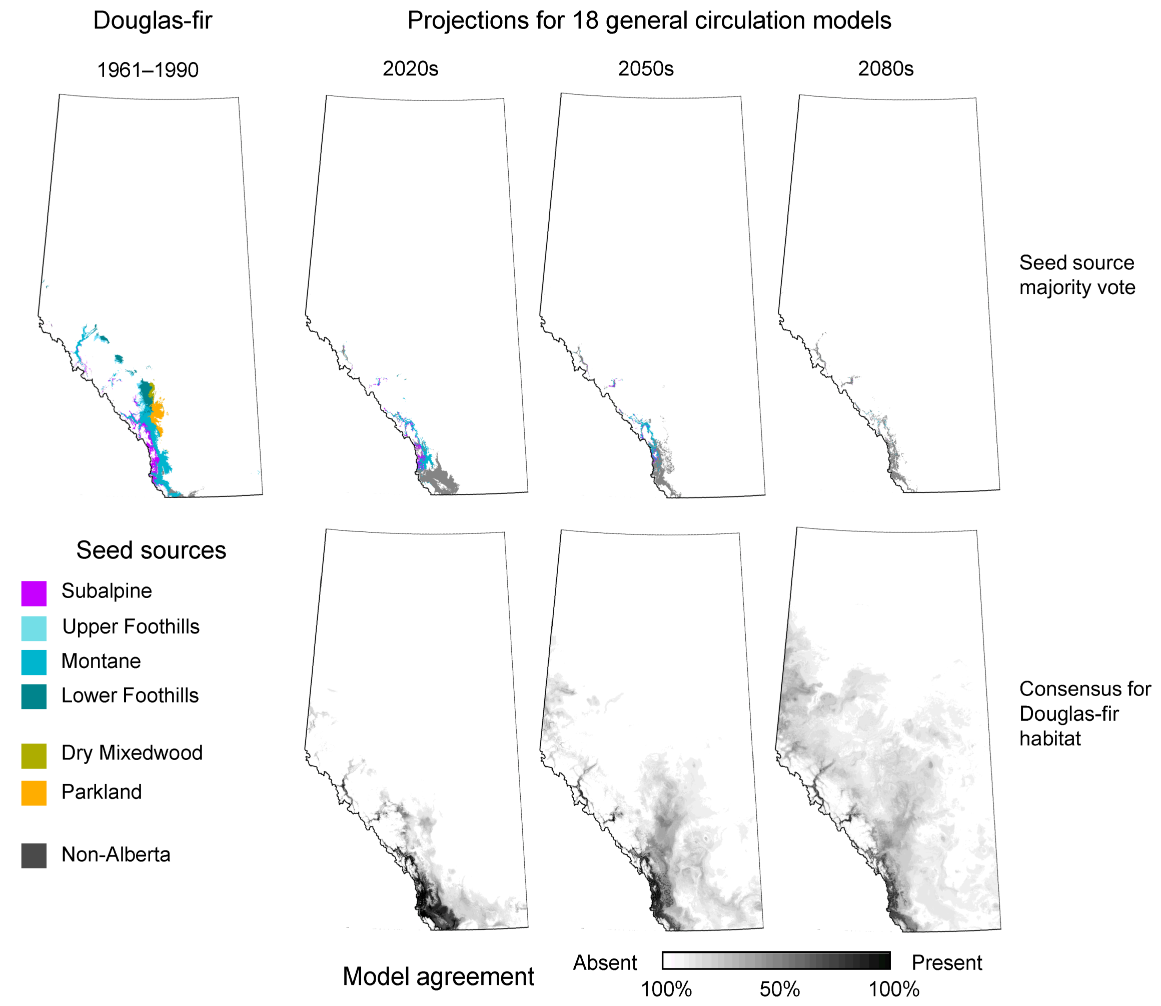

Figure S2. Seed zones projections and consensus of habitat maintenance under projected climate change for Douglas-fir in Alberta. Colors represent broad seed sources corresponding to Natural Subregions (upper row), and the gray scale represents the consensus that habitat is maintained for Douglas-fir under 18 climate change scenarios for the 2020s, 2050s, 2080s (lower row). We require at least a 70% probability that habitat is maintained to make a seed source recommendation.
