# Supplementary material for: Strategies for Reforestation under Uncertain Future Climates: Guidelines for Alberta, Canada
Source: PLoS One. 2011 Aug 10;6(8):e22977. doi: 10.1371/journal.pone.0022977 (PMC3154268; doi:10.1371/journal.pone.0022977)
Supplement: Figure S3 — Seed zones projections and consensus of habitat maintenance under projected climate change for logepole pine in Alberta. Colors represent broad seed sources corresponding to Natural Subregions (upper row), and the gray scale represents the consensus that habitat is maintained for lofgepole under 18 climate change scenarios for the 2020s, 2050s, 2080s (lower row). We require at least a 70% probability that habitat is maintained to make a seed source recommendation. (PDF) [file pone.0022977.s003.pdf]

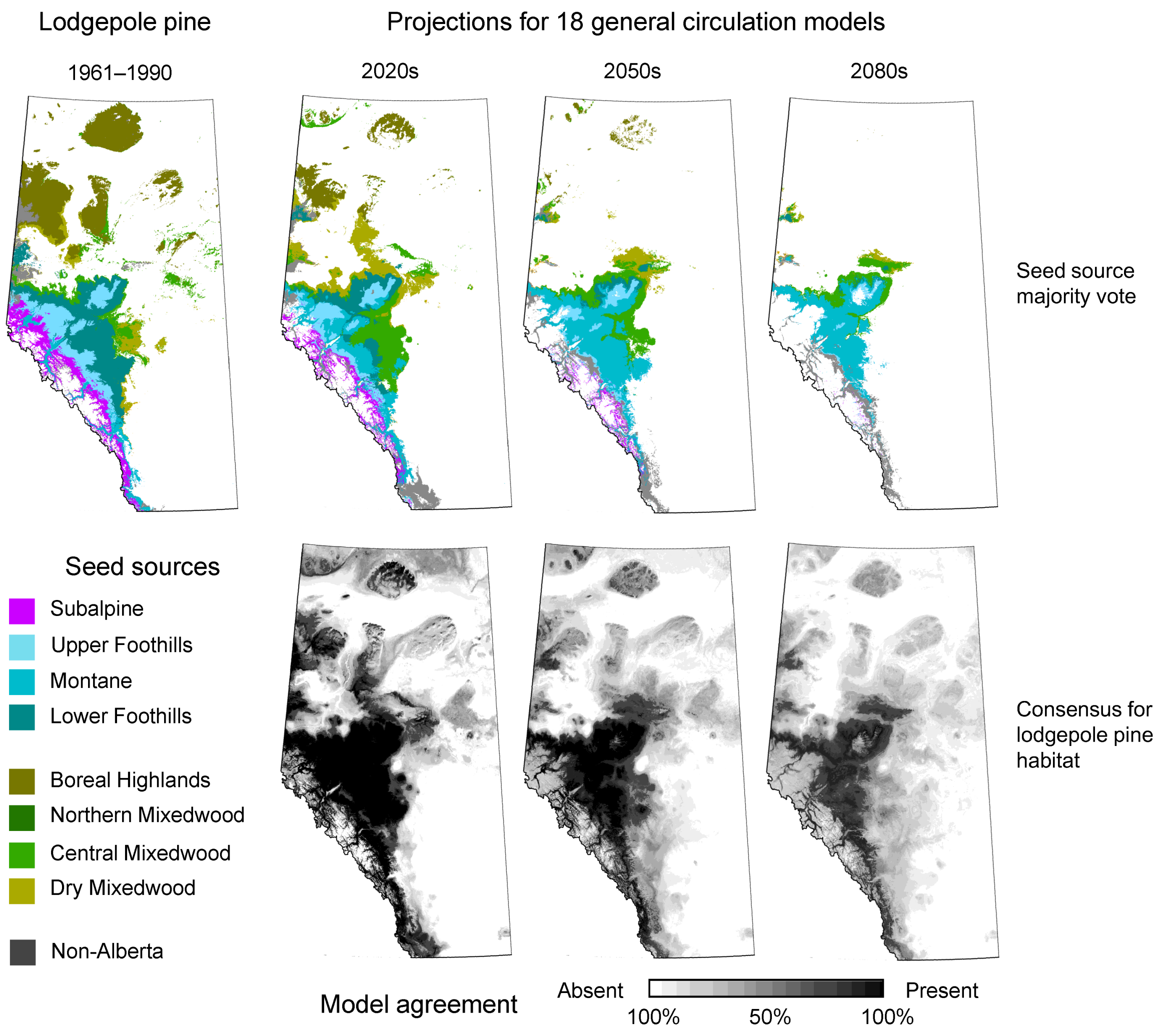

Figure S3. Seed zones projections and consensus of habitat maintenance under projected climate change for logepole pine in Alberta. Colors represent broad seed sources corresponding to Natural Subregions (upper row), and the gray scale represents the consensus that habitat is maintained for lofgepole under 18 climate change scenarios for the 2020s, 2050s, 2080s (lower row). We require at least a 70% probability that habitat is maintained to make a seed source recommendation.
