# Supplementary material for: Strategies for Reforestation under Uncertain Future Climates: Guidelines for Alberta, Canada
Source: PLoS One. 2011 Aug 10;6(8):e22977. doi: 10.1371/journal.pone.0022977 (PMC3154268; doi:10.1371/journal.pone.0022977)
Supplement: Figure S4 — Seed zones projections and consensus of habitat maintenance under projected climate change for jack pine in Alberta. Colors represent broad seed sources corresponding to Natural Subregions (upper row), and the gray scale represents the consensus that habitat is maintained for jack pine under 18 climate change scenarios for the 2020s, 2050s, 2080s (lower row). We require at least a 70% probability that habitat is maintained to make a seed source recommendation. (PDF) [file pone.0022977.s004.pdf]

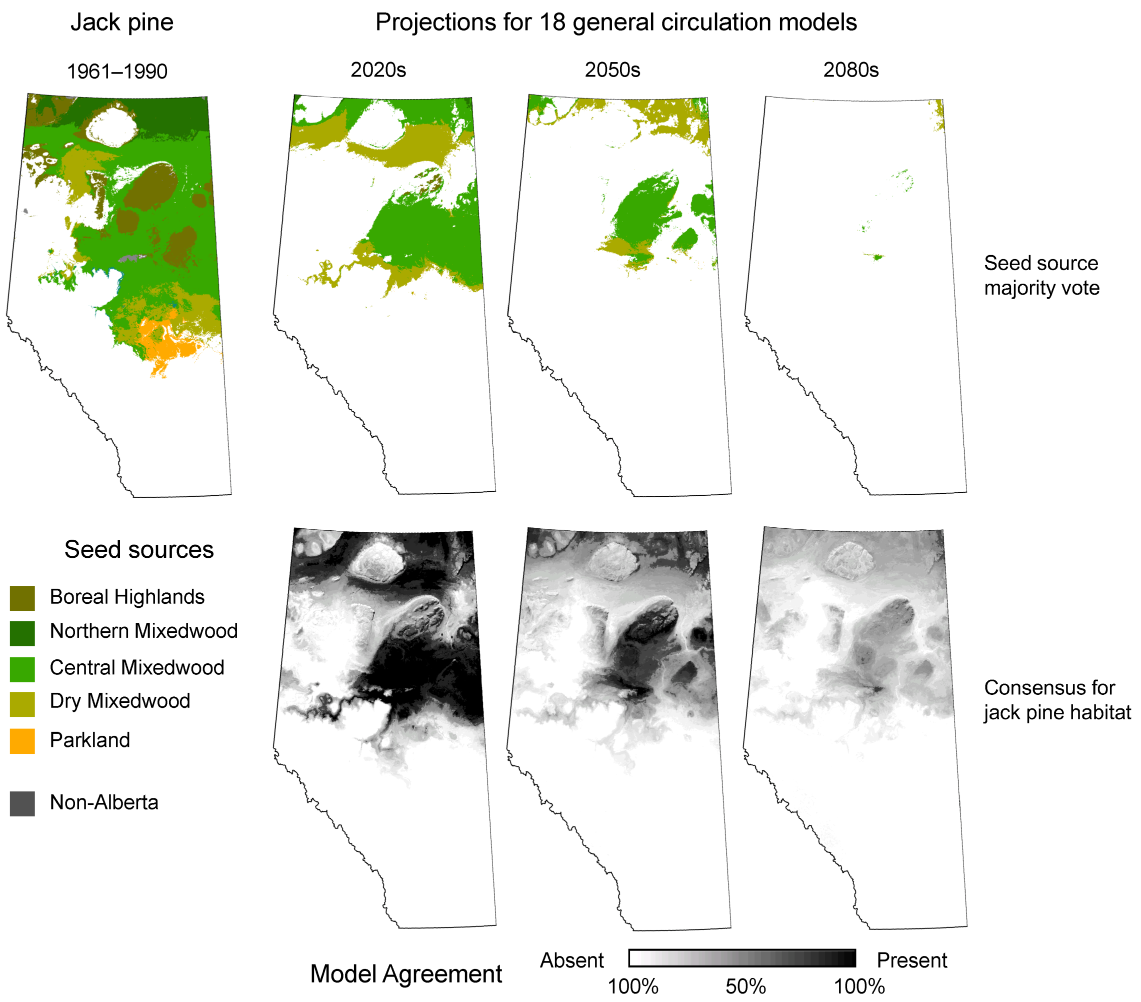

Figure S4. Seed zones projections and consensus of habitat maintenance under projected climate change for jack pine in Alberta. Colors represent broad seed sources corresponding to Natural Subregions (upper row), and the gray scale represents the consensus that habitat is maintained for jack pine under 18 climate change scenarios for the 2020s, 2050s, 2080s (lower row). We require at least a 70% probability that habitat is maintained to make a seed source recommendation.
