# Supplementary material for: Strategies for Reforestation under Uncertain Future Climates: Guidelines for Alberta, Canada
Source: PLoS One. 2011 Aug 10;6(8):e22977. doi: 10.1371/journal.pone.0022977 (PMC3154268; doi:10.1371/journal.pone.0022977)
Supplement: Table S1 — Suitable black spruce habitat expressed as % area of seed zone for observed climate, and expressed as probability of habitat maintenance under climate change projections from 18 general circulation models. (PDF) [file pone.0022977.s005.pdf]

Table S1. Suitable habitat expressed as % area of seed zone for observed climate, and expressed as probability of habitat maintenance under climate change projections from 18 general circulation models.

Black spruce	Observed Climate		Projected Climate		
seedzones*	1961-1990	1997-2006	2020s	2050s	2080s
BSA 1.1	98%	77%	96%	98%	89%
BSA 1.2	100%	100%	100%	100%	90%
CM 1.1	100%	100%	100%	94%	52%
CM 1.2	100%	100%	100%	75%	34%
CM 1.3	100%	100%	100%	95%	46%
CM 2.1	100%	99%	98%	58%	29%
CM 2.2	100%	100%	97%	60%	30%
CM 2.3	100%	100%	97%	47%	22%
CM 2.4	100%	100%	87%	52%	30%
CM 3.1	99%	82%	59%	29%	17%
CM 3.2	100%	64%	75%	42%	23%
CM 3.3	100%	100%	96%	56%	33%
CM 3.4	100%	100%	76%	36%	27%
CM 3.5	68%	99%	15%	38%	21%
DM 1.1	100%	100%	99%	62%	29%
DM 1.2	99%	83%	73%	22%	7%
DM 1.3	100%	95%	40%	11%	4%
DM 2.2	99%	26%	24%	19%	14%
DM 2.3	100%	87%	62%	28%	8%
LBH 1.1	100%	100%	100%	98%	60%
LBH 1.2	98%	88%	100%	100%	76%
LBH 1.3	100%	94%	100%	97%	61%
LBH 1.4	100%	100%	100%	77%	40%
LBH 1.5	100%	100%	99%	71%	45%
LBH 1.6	100%	100%	98%	70%	39%
LBH 2.1	100%	100%	100%	100%	83%
LF 1.1	95%	100%	100%	94%	65%
LF 1.2	95%	100%	82%	25%	20%
LF 1.3	100%	81%	84%	77%	65%
LF 1.4	100%	57%	88%	78%	59%
LF 1.5	100%	99%	43%	57%	40%
LF 2.1	100%	67%	83%	77%	57%
LF 2.2	100%	87%	74%	61%	32%
LF 2.3	81%	21%	72%	37%	14%
NM 1.1	100%	100%	100%	100%	71%
NM 2.1	99%	100%	100%	100%	81%
UBH 1.1	99%	97%	99%	100%	77%
UBH 1.2	100%	100%	100%	95%	62%
UBH 1.3	94%	100%	100%	91%	61%
UF 1.1	100%	81%	100%	93%	58%
UF 1.2	100%	57%	100%	95%	77%
UF 1.3	93%	36%	98%	79%	44%
UF 1.4	98%	72%	99%	88%	60%
UF 1.5	77%	63%	78%	68%	29%
UF 2.4	100%	99%	96%	93%	67%
UF 2.5	65%	90%	86%	86%	53%
