# Supplementary material for: Strategies for Reforestation under Uncertain Future Climates: Guidelines for Alberta, Canada
Source: PLoS One. 2011 Aug 10;6(8):e22977. doi: 10.1371/journal.pone.0022977 (PMC3154268; doi:10.1371/journal.pone.0022977)
Supplement: Table S2 — Suitable Douglas-fir habitat expressed as % area of seed zone for observed climate, and expressed as probability of habitat maintenance under climate change projections from 18 general circulation models. (PDF) [file pone.0022977.s006.pdf]

Table S2. Suitable habitat expressed as % area of seed zone for observed climate, and expressed as probability of habitat maintenance under climate change projections from 18 general circulation models.

Douglas-fir	Observed Climate		Projected Climate		
seedzones*	1961-1990	1997-2006	2020s	2050s	2080s
M 2.2	91%	70%	55%	57%	64%
M 4.3	97%	56%	39%	53%	50%
M 4.5	100%	100%	78%	73%	51%
M 5.3	77%	38%	49%	50%	57%
M 5.5	100%	100%	88%	71%	56%
M 5.6	85%	87%	85%	78%	72%
