# Supplementary material for: Strategies for Reforestation under Uncertain Future Climates: Guidelines for Alberta, Canada
Source: PLoS One. 2011 Aug 10;6(8):e22977. doi: 10.1371/journal.pone.0022977 (PMC3154268; doi:10.1371/journal.pone.0022977)
Supplement: Table S3 — Suitable lodgepole pine habitat expressed as % area of seed zone for observed climate, and expressed as probability of habitat maintenance under climate change projections from 18 general circulation models. (PDF) [file pone.0022977.s007.pdf]

Table S3. Suitable habitat expressed as % area of seed zone for observed climate, and expressed as probability of habitat maintenance under climate change projections from 18 general circulation models.

Lodgepole Pine	Observed Climate		Projected Climate		
seedzones*	1961-1990	1997-2006	2020s	2050s	2080s
BSA 1.1	98%	59%	67%	61%	47%
CM 3.2	48%	15%	70%	55%	45%
CM 3.3	21%	71%	74%	68%	58%
CM 3.4	100%	86%	87%	72%	69%
CM 3.5	95%	7%	94%	76%	49%
LBH 1.2	96%	20%	46%	34%	17%
LBH 1.5	99%	44%	42%	40%	24%
LBH 2.1	100%	89%	65%	49%	24%
LF 1.2	100%	100%	88%	59%	66%
LF 1.3	100%	75%	100%	99%	87%
LF 1.4	100%	96%	99%	98%	85%
LF 1.5	100%	54%	99%	91%	69%
LF 2.1	100%	99%	98%	98%	88%
LF 2.2	100%	99%	89%	86%	61%
LF 2.3	100%	100%	65%	51%	51%
M 1.1	99%	75%	27%	23%	11%
M 2.1	100%	100%	99%	93%	67%
M 2.2	100%	89%	70%	63%	61%
M 3.2	100%	98%	79%	80%	76%
M 4.1	100%	100%	86%	85%	78%
M 4.2	100%	100%	99%	87%	91%
M 4.3	100%	100%	55%	57%	51%
M 4.4	100%	100%	60%	58%	48%
M 4.5	98%	98%	78%	73%	51%
M 5.1	100%	95%	85%	80%	79%
M 5.3	100%	100%	89%	81%	71%
M 5.4	100%	100%	96%	75%	63%
M 5.5	100%	100%	88%	72%	56%
M 5.6	96%	86%	90%	82%	71%
UBH 1.2	87%	97%	54%	43%	39%
UBH 1.3	93%	100%	80%	76%	64%
UF 1.1	100%	100%	100%	93%	67%
UF 1.2	100%	100%	100%	100%	89%
UF 1.3	100%	91%	98%	84%	56%
UF 1.4	100%	100%	97%	95%	85%
UF 1.5	100%	100%	91%	77%	61%
UF 2.4	100%	100%	99%	92%	90%
UF 2.5	100%	100%	100%	90%	86%
