# Supplementary material for: Strategies for Reforestation under Uncertain Future Climates: Guidelines for Alberta, Canada
Source: PLoS One. 2011 Aug 10;6(8):e22977. doi: 10.1371/journal.pone.0022977 (PMC3154268; doi:10.1371/journal.pone.0022977)
Supplement: Table S4 — Suitable jack pine habitat expressed as % area of seed zone for observed climate, and expressed as probability of habitat maintenance under climate change projections from 18 general circulation models. (PDF) [file pone.0022977.s008.pdf]

Table S4. Suitable habitat expressed as % area of seed zone for observed climate, and expressed as probability of habitat maintenance under climate change projections from 18 general circulation models.

Jack Pine	Observed Climate		Projected Climate		
seedzones*	1961-1990	1997-2006	2020s	2050s	2080s
CM 1.1	99%	100%	89%	52%	31%
CM 1.3	94%	87%	71%	32%	15%
CM 2.1	100%	36%	69%	55%	22%
CM 2.2	100%	10%	84%	71%	33%
CM 3.1	97%	20%	72%	31%	14%
CM 3.2	96%	16%	73%	37%	16%
CM 3.3	88%	37%	67%	42%	21%
DM 1.1	89%	41%	38%	10%	3%
LBH 1.5	100%	54%	97%	70%	38%
LF 1.1	81%	41%	86%	73%	47%
NM 1.1	98%	91%	85%	69%	43%
NM 2.1	93%	42%	57%	74%	42%
UBH 1.2	78%	17%	23%	44%	34%
