# Supplementary material for: Strategies for Reforestation under Uncertain Future Climates: Guidelines for Alberta, Canada
Source: PLoS One. 2011 Aug 10;6(8):e22977. doi: 10.1371/journal.pone.0022977 (PMC3154268; doi:10.1371/journal.pone.0022977)
Supplement: Table S5 — Suitable white spruce habitat expressed as % area of seed zone for observed climate, and expressed as probability of habitat maintenance under climate change projections from 18 general circulation models. (PDF) [file pone.0022977.s009.pdf]

Table S5. Suitable habitat expressed as % area of seed zone for observed climate, and expressed as probability of habitat maintenance under climate change projections from 18 general circulation models.

White spruce	Observed Climate		Projected Climate		
seedzones*	1961-1990	1997-2006	2020s	2050s	2080s
BSA 1.1	98%	77%	96%	98%	90%
BSA 1.2	99%	100%	100%	100%	91%
CM 1.1	100%	100%	100%	98%	75%
CM 1.2	100%	100%	100%	92%	67%
CM 1.3	100%	100%	100%	98%	71%
CM 2.1	100%	100%	100%	88%	79%
CM 2.2	100%	100%	100%	92%	80%
CM 2.3	100%	100%	100%	82%	69%
CM 2.4	100%	99%	96%	85%	77%
CM 3.1	99%	91%	85%	83%	77%
CM 3.2	100%	97%	84%	81%	77%
CM 3.3	100%	100%	98%	88%	74%
CM 3.4	100%	100%	95%	95%	78%
CM 3.5	100%	100%	100%	91%	71%
DM 1.1	100%	100%	99%	85%	56%
DM 1.2	99%	98%	88%	66%	50%
DM 1.3	100%	100%	74%	74%	59%
DM 2.1	73%	95%	74%	88%	57%
DM 2.2	99%	99%	67%	87%	69%
DM 2.3	100%	85%	87%	71%	58%
LBH 1.1	100%	100%	100%	99%	76%
LBH 1.2	98%	88%	100%	100%	86%
LBH 1.3	100%	98%	100%	99%	82%
LBH 1.4	100%	100%	100%	89%	69%
LBH 1.5	100%	100%	100%	79%	81%
LBH 1.6	100%	100%	99%	88%	69%
LBH 2.1	100%	99%	100%	100%	86%
LF 1.1	92%	100%	100%	98%	89%
LF 1.2	100%	100%	95%	95%	74%
LF 1.3	100%	99%	100%	100%	88%
LF 1.4	100%	100%	100%	98%	80%
LF 1.5	100%	100%	100%	98%	79%
LF 2.1	100%	99%	100%	99%	80%
LF 2.2	100%	100%	100%	100%	76%
LF 2.3	100%	100%	78%	70%	50%
M 1.1	100%	52%	25%	3%	1%
M 2.1	100%	100%	92%	54%	33%
M 2.2	100%	49%	69%	56%	30%
M 3.2	100%	57%	80%	67%	41%
M 4.1	100%	52%	83%	71%	42%
M 4.2	100%	100%	100%	97%	68%
M 4.3	98%	66%	54%	41%	30%
M 5.1	98%	34%	74%	59%	36%
M 5.3	98%	64%	85%	67%	44%
M 5.4	47%	77%	95%	69%	51%
NM 1.1	100%	100%	100%	100%	87%
NM 2.1	99%	100%	100%	100%	85%
UBH 1.1	99%	98%	99%	100%	82%
UBH 1.2	100%	100%	100%	96%	80%
UBH 1.3	94%	100%	100%	97%	86%
UF 1.1	100%	100%	100%	93%	65%
UF 1.2	100%	100%	100%	100%	89%
UF 1.3	100%	83%	98%	79%	54%
UF 1.4	100%	100%	100%	99%	77%
UF 1.5	100%	100%	97%	87%	63%
UF 2.4	100%	100%	100%	100%	80%
UF 2.5	99%	96%	100%	94%	67%
