# Supplementary material for: Strategies for Reforestation under Uncertain Future Climates: Guidelines for Alberta, Canada
Source: PLoS One. 2011 Aug 10;6(8):e22977. doi: 10.1371/journal.pone.0022977 (PMC3154268; doi:10.1371/journal.pone.0022977)
Supplement: Table S6 — Table of best matching seed sources for 1961–1990 climate. The multivariate Mahalanobis climate distance is given in parenthesis. (PDF) [file pone.0022977.s010.pdf]

Table S6. Table of best matching seed sources for 1961-1990 climate. The multivariate Mahalanobis climate distance is given in parenthesis.

Seed Zone	Choice 1	Choice 2	Choice 3	Choice 4	Choice 5	Choice 6	Choice 7	Choice 8	Choice 9	Choice 10
Northern Mixedwood										
NM11	NM11(0)	KU11(0.5)	LBH12(1.2)	NM21(2.3)	LBH21(2.6)	PAD11(2.9)	CM11(3.2)			
NM21	NM21(0)	LBH21(0.3)	BSA12(0.8)	LBH12(1)	KU11(1.7)	NM11(2.3)	LBH11(3.2)			
Central Mixedwood										
CM11	CM11(0)	PAD11(0)	AP11(0.1)	CM13(0.6)	LBH11(0.8)	CM12(1.7)	DM11(2)	LBH12(2.6)	CM21(2.9)	KU11(2.9)
CM12	CM12(0)	CM21(0.4)	DM11(0.4)	CM22(1.2)	UBH12(1.2)	AP11(1.4)	CM13(1.4)	LBH14(1.4)	CM11(1.7)	
CM13	CM13(0)	LBH11(0.5)	AP11(0.6)	CM11(0.6)	PAD11(0.6)	CM21(2)	DM11(1)	CM12(1.4)	UBH12(2)	LBH14(2.9)
CM21	CM21(0)	CM12(0.4)	CM22(0.5)	DM11(0.6)	LBH14(0.8)	UBH12(1)	CM23(1.2)	CM24(1.4)	LBH13(1.7)	LBH15(1.7)
CM22	CM22(0)	CM23(0.4)	CM21(0.5)	LBH14(0.5)	CM24(1)	LBH13(1)	CM12(1.2)	UBH12(1.2)	DM11(1.7)	LBH15(2)
CM23	CM23(0)	CM22(0.4)	CM24(0.8)	LBH14(0.8)	CM21(1.2)	LBH13(1.7)	CM12(2)	UBH12(2)	DM11(2.6)	LBH15(2.6)
CM24	CM24(0)	CM23(0.8)	LBH15(0.8)	LF11(0.8)	CM22(1)	CM21(1.4)	CM31(1.7)	LBH14(1.7)		
CM31	CM31(0)	CM33(0.5)	LBH15(0.6)	DM21(0.8)	LF11(1)	CM32(1.2)	DM13(1.2)	DM12(1.4)	PRP11(1.4)	
CM32	CM32(0)	DM22(0.5)	CM33(0.8)	CM34(0.8)	CP11(0.8)	DM21(0.8)	CM31(1.2)	CP12(2)	DM13(2)	LF12(2)
CM33	CM33(0)	CM31(0.5)	DM13(0.6)	LF11(0.6)	CM32(0.8)	CM34(0.8)	DM21(0.8)	PRP11(1.2)	CP11(1.7)	CP12(1.7)
CM34	CM34(0)	CM32(0.8)	CM33(0.8)	CP11(1)	CP12(1)	DM22(1)	DM21(1.2)	LF12(1.2)	DM13(1.4)	DM23(1.7)
CM35	CM35(0)	LF15(0.2)	LF14(0.5)	LF13(1)	DM22(1.2)	DM23(1.4)	LF21(1.4)	UF12(1.7)	LF22(2)	
Dry Mixedwood										
DM11	DM11(0)	CM12(0.4)	CM21(0.6)	CM13(1)	LBH14(1.4)	UBH12(1.4)	AP11(1.7)	CM22(1.7)	CM11(2)	LBH16(2)
DM12	DM12(0)	DM13(0.8)	LBH16(0.8)	PRP11(0.8)	CM31(1.4)	LBH15(1.7)	CM33(2)	UBH13(2)	CM24(2.3)	DM21(2.3)
DM13	DM13(0)	PRP11(0.3)	CM33(0.6)	DM12(0.8)	DM21(1)	CM31(1.2)	CP12(1.2)	CM34(1.4)	CP11(1.7)	LF12(1.7)
DM21	DM21(0)	CP11(0.4)	CM31(0.8)	CM32(0.8)	CM33(0.8)	CP12(0.8)	DM13(1)	DM22(1)	PRP11(1)	
DM22	DM22(0)	CP11(0.4)	CM32(0.5)	CM34(1)	DM21(1)	CM35(1.2)	CP12(1.4)	LF12(1.7)	LF14(1.7)	LF15(1.7)
DM23	DM23(0)	LF21(0.5)	LF22(0.5)	LF15(0.8)	LF14(1)	CM35(1.4)	UF14(1.4)	CM34(1.7)		
Boreal Highlands										
BSA11	BSA11(0)	BSA12(2.6)	LBH12(2.6)	LBH21(2.6)						
BSA12	BSA12(0)	LBH21(0.6)	NM21(0.8)	LBH12(2)	BSA11(2.6)	KU11(2.9)				
LBH11	LBH11(0)	CM13(0.5)	AP11(0.8)	CM11(0.8)	PAD11(1)	UBH12(2)	CM12(2.3)	DM11(2.3)		
LBH12	LBH12(0)	LBH21(0.6)	KU11(1)	NM21(1)	NM11(1.2)	BSA12(2)				
LBH13	LBH13(0)	UBH11(0.8)	LBH14(1.2)	UBH12(1.2)	CM21(1.7)	CM23(1.7)	CM22(1)	CM12(2.3)	LBH11(3.2)	
LBH14	LBH14(0)	UBH12(0.4)	CM22(0.5)	CM21(0.8)	CM23(0.8)	LBH13(1.2)	CM12(1.4)	DM11(1.4)	LBH16(1.4)	
LBH15	LBH15(0)	CM31(0.6)	CM24(0.8)	LF11(1)	LBH16(1.2)	CM21(1.7)	CM33(1.7)	DM12(1.7)	CM22(2)	LBH14(2)
LBH16	LBH16(0)	DM12(0.8)	UBH13(1)	LBH15(1.2)	LBH14(1.4)	UBH12(1.4)	CM31(2)	DM11(2)	CM21(2.3)	CM24(2.3)
LBH21	LBH21(0)	NM21(0.3)	BSA12(0.6)	LBH12(0.6)	KU11(2)	BSA11(2.6)	NM11(2.6)	LBH11(3.2)		
UBH11	UBH11(0)	LBH13(0.8)	UBH12(1.7)	LBH11(2.6)	LBH14(2.6)	CM21(3.2)	CM22(3.2)	CM12(3.6)		
UBH12	UBH12(0)	LBH14(0.4)	CM21(1)	CM12(1.2)	CM22(1.2)	LBH13(1.2)	DM11(1.4)	LBH16(1.4)	UBH11(1.7)	
UBH13	UBH13(0)	LBH16(1)	DM12(2)	LBH15(2.3)	PRP11(2.3)	CM31(2.6)	LF12(2.6)	DM13(2.9)	DM21(3.2)	
Lower Foothills										
LF11	LF11(0)	CM33(0.6)	CM24(0.8)	CM31(1)	LBH15(1)	DM13(2)	CM32(2.3)	CM34(2.6)	DM12(2.6)	
LF12	LF12(0)	CP11(1)	CP12(1)	CM34(1.2)	DM21(1.2)	PRP11(1.2)	BWBSmw1(	DM13(1.7)	DM22(1.7)	NF11(1.7)
LF13	LF13(0)	CM35(1)	UF12(1)	LF14(1.2)	LF15(1.4)	BWBSwk1(2	DM22(2.9)	LF21(2.9)	UF13(2.9)	
LF14	LF14(0)	LF15(0.4)	CM35(0.5)	LF21(0.6)	UF12(0.6)	DM23(1)	LF13(1.2)	LF22(1.4)	UF14(1.4)	DM22(1.7)
LF15	LF15(0)	CM35(0.2)	LF14(0.4)	LF21(0.5)	DM23(0.8)	LF22(1)	UF12(1.2)	LF13(1.4)		
LF21	LF21(0)	LF22(0.3)	DM23(0.5)	LF15(0.5)	LF14(0.6)	UF14(0.8)	UF12(1.2)	CM35(1.4)	UF13(2.3)	
LF22	LF22(0)	LF21(0.3)	DM23(0.5)	LF15(1)	UF14(1.2)	LF14(1.4)	CM35(2)	LF23(2.3)	CM34(2.6)	UF12(2.6)
LF23	LF23(0)	UF15(0.3)	FP11(1)	M44(1.4)	M43(1.7)	LF22(2.3)	M53(2.3)	UF25(2.3)	M32(2.6)	
Montane										
M11	M11(0)	MG13(2.3)								
M21	M21(0)	UF13(0.8)	UF14(2)	UF24(2)	SA11(2.6)	UF12(2.6)	LF14(3.6)			
M22	M22(0)	M32(0.5)	FF11(1)	M41(1.2)	M45(1.2)	FP11(1.4)	M51(1.7)	M55(1.7)	MG11(1.7)	LF12(2.3)
M32	M32(0)	M22(0.5)	M41(0.8)	M45(1)	UF25(1.2)	FF11(1.4)	FP11(1.4)	M51(1.4)	M55(1.4)	
M41	M41(0)	M32(0.8)	M51(0.8)	UF25(0.8)	M53(1)	M54(1)	M22(1.2)	M45(1.2)	M55(1.4)	M44(1.7)
M42	M42(0)	SA31(1.2)	UF25(2)	M53(2.6)	M54(2.9)	UF15(2.9)	M41(3.2)	SA41(3.6)		
M43	M43(0)	M44(0.1)	FP11(0.5)	M53(0.5)	M54(0.6)	UF15(1)	LF23(1.7)	FF11(2)	M41(2)	UF25(2)
M44	M44(0)	M43(0.1)	FP11(0.5)	M53(0.5)	M54(0.5)	UF15(0.8)	LF23(1.4)	M41(1.7)	UF25(1.7)	FF11(2)
M45	M45(0)	M55(0.4)	M51(1)	M32(1)	FF11(1.2)	M22(1.2)	M41(1.2)	M54(1.2)	FP12(1.4)	M56(1.4)
M51	M51(0)	M55(0.6)	M41(0.8)	M45(1)	M54(1.2)	M32(1.4)	M53(1.4)	SA32(1.4)	M22(1.7)	UF25(1.7)
M53	M53(0)	M54(0.2)	M43(0.5)	M44(0.5)	UF25(0.8)	M41(1)	UF15(1)	FP11(1.2)	M51(1.4)	M45(1.7)
M54	M54(0)	M53(0.2)	M44(0.5)	M43(0.6)	M41(1)	M45(1.2)	M51(1.2)	UF25(1.2)	FP11(1.4)	UF15(1.7)
M55	M55(0)	M45(0.4)	M51(0.6)	M56(1)	M32(1.4)	M41(1.4)	M22(1.7)	MSdk(1.7)	SA32(1.7)	
M56	M56(0)	SA33(0.8)	M55(1)	FP12(1.4)	M45(1.4)					
Upper Foothills										
UF11	UF11(0)									
UF12	UF12(0)	LF14(0.6)	UF13(0.8)	LF13(1)	LF15(1.2)	LF21(1.2)	UF14(1.4)	CM35(1.7)	DM23(2.3)	
UF13	UF13(0)	M21(0.8)	UF12(0.8)	UF14(1)	UF24(1.4)	LF14(1.7)	LF21(2.3)	SA11(2.3)	DM23(2.9)	
UF14	UF14(0)	LF21(0.8)	UF24(0.8)	UF13(1)	LF22(1.2)	DM23(1.4)	LF14(1.4)	UF12(1.4)	LF15(1.7)	
UF15	UF15(0)	LF23(0.3)	FP11(0.8)	M44(0.8)	M43(1)	M53(1)	UF25(1.2)	M54(1.7)	M32(2)	
UF24	UF24(0)	UF14(0.8)	SA11(1.4)	UF13(1.4)	UF25(1.4)	M32(1.7)	SA12(1.7)	M21(2)	M41(2)	LF21(2.6)
UF25	UF25(0)	M41(0.8)	M53(0.8)	M32(1.2)	M54(1.2)	UF15(1.2)	SA31(1.4)	UF24(1.4)		
