# Supplementary material for: Strategies for Reforestation under Uncertain Future Climates: Guidelines for Alberta, Canada
Source: PLoS One. 2011 Aug 10;6(8):e22977. doi: 10.1371/journal.pone.0022977 (PMC3154268; doi:10.1371/journal.pone.0022977)
Supplement: Table S7 — Table of best matching seed sources for 1997–2006 climate. The multivariate Mahalanobis climate distance is given in parenthesis. (PDF) [file pone.0022977.s011.pdf]

Table S7. Table of best matching seed sources for 1997-2006 climate. The multivariate Mahalanobis climate distance is given in parenthesis.

Seed Zone	Choice 1	Choice 2	Choice 3	Choice 4	Choice 5	Choice 6	Choice 7	Choice 8	Choice 9	Choice 10
Northern Mixedwood										
NM11	CM11(4.4)	PAD11(4.4)	AP11(4.8)							
NM21	AP11(0.6)	CM11(0.6)	PAD11(0.8)	LBH11(1.4)	CM13(1.7)	CM12(2)	CM21(2.6)	UBH12(2.6)	LBH12(2.6)	
Central Mixedwood										
CM11	CM12(3.6)	DM11(4)	CM21(4.8)							
CM12	CM12(3.2)	CM24(3.2)	CM21(3.6)	CM23(3.6)	DM11(4)	CM31(4)	LBH15(4)	CM22(4)	LBH16(4.4)	DM12(4.4)
CM13	CM12(1.2)	DM11(1.4)	CM21(1.4)	CM22(2)	CM23(2)	LBH14(2.3)	CM24(2.3)	LBH16(2.6)	UBH12(2.6)	LBH15(2.6)
CM21	CM31(2.6)	LBH15(2.9)	CM24(2.9)	LF11(3.6)	CM21(4)	DM12(4)	CM33(4)	CM12(4.4)	DM11(4.4)	LBH16(4.4)
CM22	CM24(2)	LBH15(2.6)	CM23(2.6)	CM31(2.9)	LF11(2.9)	CM21(3.2)	CM22(3.2)	CM12(3.6)	LBH16(3.6)	DM12(3.6)
CM23	CM24(2.6)	CM23(2.6)	CM22(3.6)	LBH15(4)	LF11(4)	CM21(4.4)	LBH14(4.4)	DM12(4.4)	CM12(4.8)	LBH16(4.8)
CM24	LF11(2.3)	CM31(2.6)	CM24(2.6)	CM33(2.9)	LBH15(3.2)	DM12(3.6)	DM21(3.6)	DM13(3.6)	PRP11(4)	CM32(4)
CM31	DM21(1.7)	CM31(2.3)	CM33(2.3)	CM32(2.3)	CP11(2.3)	PRP11(2.9)	DM13(2.9)	CP12(2.9)	DM22(2.9)	LF11(3.2)
CM32	DM21(1.7)	CM32(1.7)	CP11(1.7)	DM22(1.7)	CM34(2)	CM33(2.3)	CP12(2.3)	DM13(2.6)	PRP11(2.9)	LF12(2.9)
CM33	CM33(2)	DM13(2)	LF11(2.6)	DM21(2.6)	PRP11(2.6)	CP12(2.6)	CM34(2.6)	CM31(2.9)	CM32(2.9)	NF11(2.9)
CM34	CM34(1.4)	CP12(1.7)	DM13(2.3)	M22(2.3)	M32(2.3)	MG11(2.3)	CM33(2.6)	PRP11(2.6)	LF12(2.6)	NF11(2.6)
CM35	CM35(0.8)	LF15(0.8)	LF14(1)	LF21(1.2)	LF13(1.2)	DM23(1.7)	DM22(2)	LF22(2)	UF14(2.3)	UF13(2.6)
Dry Mixedwood										
DM11	CM24(2.3)	CM23(2.6)	CM12(2.9)	DM11(3.2)	CM21(3.2)	LBH15(3.2)	CM31(3.2)	CM22(3.2)	DM12(3.2)	LBH16(3.6)
DM12	DM12(2.3)	PRP11(2.6)	DM13(2.6)	CM31(3.6)	CM33(3.6)	NF11(3.6)	LBH16(4)	CM24(4)	LF11(4)	DM21(4)
DM13	PRP11(2)	DM13(2)	NF11(2.3)	MG11(2.3)	CP12(2.6)	DM12(2.9)	CM33(2.9)	DMG11(2.9)	M22(2.9)	
DM21	CP11(1.4)	DM21(1.7)	CP12(2)	DM22(2)	CM32(2.3)	NF11(2.3)	CM34(2.6)	CM33(2.9)	PRP11(2.9)	DM13(2.9)
DM22	DM22(1.2)	CP11(1.4)	CP12(2)	CM34(2)	DM21(2.3)	CM32(2.3)	LF14(2.3)	CM35(2.3)	LF12(2.6)	LF15(2.6)
DM23	LF21(0.8)	DM23(1)	LF15(1)	UF14(1.2)	LF22(1.2)	LF14(1.4)	CM35(1.4)	UF12(1.4)	UF13(2)	LF13(2.6)
Boreal Highlands										
BSA11	BSA11(4.4)	UBH11(4.8)								
BSA12	LBH12(1.2)	UBH12(4)	CM11(2.3)	KU11(2.3)	PAD11(2.3)	LBH11(2.6)	LBH21(2.9)	NM11(2.9)	UBH11(3.2)	BSA11(3.6)
LBH11	CM12(0.8)	CM21(1)	UBH12(1.2)	LBH14(1.2)	CM22(1.2)	CM23(1.4)	DM11(1.7)	LBH13(2)		
LBH12	CM11(2.9)	PAD11(3.2)	CM12(4)	UBH12(4)	CM13(4)	LBH11(4.4)	LBH12(4.4)	DM11(4.8)	CM21(4.8)	NM11(4.8)
LBH13	CM23(2.3)	CM22(2.6)	CM21(2.9)	UBH12(2.9)	LBH14(2.9)	CM24(2.9)	CM12(3.2)	LBH13(3.2)	LBH15(3.6)	
LBH14	CM23(1.7)	CM24(2.3)	LBH14(2.6)	LBH16(2.9)	CM22(2.9)	CM21(3.2)	UBH12(3.2)	LBH15(3.2)	CM12(3.6)	DM12(3.6)
LBH15	CM31(1.7)	LBH15(2.6)	LF11(2.6)	DM21(2.6)	CM33(2.6)	DM13(2.9)	CM24(3.2)	DM12(3.2)	PRP11(3.2)	CM32(3.2)
LBH16	DM12(2.3)	LBH16(2.6)	CM24(2.9)	CM31(3.2)	UBH13(3.2)	LF11(3.2)	DM13(3.2)	LBH15(3.6)	CM23(3.6)	PRP11(3.6)
LBH21	CM11(1.2)	PAD11(1.4)	LBH11(1.7)	CM12(2.3)	UBH12(2.3)	CM13(2.3)	UBH11(2.3)	LBH12(2.3)	LBH13(2.9)	
UBH11	UBH12(2.9)	LBH14(3.6)	CM12(4)	CM21(4)	LBH13(4)	UBH11(4)	LBH16(4.4)	CM22(4.4)	CM23(4.4)	LBH15(4.8)
UBH12	CM23(2.3)	UBH12(2.6)	LBH14(2.6)	CM22(2.9)	CM21(3.2)	LBH16(3.2)	CM24(3.2)	LBH13(3.2)	CM12(3.6)	LBH15(3.6)
UBH13	UBH13(2.6)	DM12(2.9)	LBH16(3.2)	LF11(3.2)	PRP11(3.2)	M41(3.2)	M51(3.2)	DM13(3.6)	M22(3.6)	M32(3.6)
Lower Foothills										
LF11	LF11(2)	CM33(2.3)	CM31(2.6)	DM13(2.9)	CM24(3.2)	DM21(3.2)	LBH15(3.6)	PRP11(3.6)	CM32(3.6)	CM34(3.6)
LF12	PRP11(2)	NF11(2)	LF12(2)	CP12(2)	DM13(2.3)	CM34(2.3)	M32(2.3)	MG11(2.3)	CP11(2.9)	FF11(2.9)
LF13	UF12(1)	LF14(1.2)	LF13(1.4)	LF21(2)	UF13(2)	LF15(2)	CM35(2.3)	DM23(2.9)	UF14(2.9)	DM22(3.2)
LF14	LF21(0.8)	LF14(1.2)	UF12(1.2)	DM23(1.4)	UF14(1.4)	UF13(1.4)	LF22(1.7)	M32(2)	LF15(2)	CM34(2.3)
LF15	LF21(0.6)	LF15(0.6)	LF14(0.8)	CM35(1)	UF12(1)	DM23(1.2)	LF22(1.4)	LF13(1.4)	UF14(1.7)	UF13(2)
LF21	LF21(0.6)	UF14(1)	LF22(1.2)	DM23(1.4)	LF14(1.7)	UF13(1.7)	UF12(1.7)	LF15(2)	M32(2.3)	UF24(2.3)
LF22	LF21(0.5)	LF22(0.6)	UF14(0.8)	DM23(1.2)	LF15(1.2)	LF14(1.7)	CM35(2)	UF12(2)	UF13(2.3)	UF24(2.9)
LF23	LF23(1.2)	UF15(1.7)	UF14(2)	LF22(2.3)	UF24(2.9)	LF21(2.9)	FP11(2.9)	DM23(3.2)	UF25(3.2)	M32(3.6)
Montane										
M11	M11(4.4)	M21(4.4)								
M21	M21(0.6)	UF24(2)	UF13(2)	UF14(2.6)	SA11(2.9)	M32(3.2)	M22(4)	M41(4)	UF12(4)	
M22	M22(0.5)	M32(0.8)	M41(1.7)	FF11(1.7)	M45(2)	FP11(2)	M55(2.3)	UF24(2.3)	M21(2.3)	M51(2.6)
M32	M32(0.4)	M22(0.8)	UF24(1)	M41(1.2)	M21(1.4)	UF25(1.7)	UF14(1.7)	M51(2)	FP11(2)	UF13(2)
M41	M41(1)	UF24(1)	M32(1.2)	M22(1.7)	M51(1.7)	UF25(1.7)	M21(2)	M55(2.3)	M45(2.3)	
M42	UF24(1.4)	SA12(2.3)	UF14(2.3)	UF25(2.6)	SA11(2.9)	UF13(2.9)	M32(3.2)	M41(3.6)	M51(3.6)	
M43	UF15(1.2)	LF23(1.2)	FP11(1.4)	M41(1.7)	UF25(1.7)	M44(1.7)	M32(2)	M53(2)	M43(2)	UF24(2)
M44	UF24(1.7)	UF15(1.7)	LF23(1.7)	UF14(2)	UF25(2.3)	M41(2.6)	M32(2.6)	M44(2.6)	FP11(2.6)	M53(2.9)
M45	UF24(2)	M21(2.3)	SA12(2.6)	M41(2.6)	M32(2.6)	M55(2.6)	M45(2.6)	SA11(2.6)		
M51	ESSFdc1(0.8)	M41(1)	M51(1)	M32(1.2)	UF24(1.2)	SA12(1.4)	M55(1.7)	UF25(1.7)	SA11(2)	M45(2)
M53	UF24(0.8)	UF25(1.4)	UF14(1.4)	M41(1.7)	M32(1.7)	UF15(2)	M53(2.3)	LF23(2.3)	M21(2.6)	M51(2.9)
M54	UF24(1)	UF25(1.7)	UF14(1.7)	M41(2)	M32(2.3)	UF15(2.6)	M21(2.6)	SA12(2.9)	M45(2.9)	M53(2.9)
M55	UF24(2)	SA12(2.3)	SA11(2.3)	M55(2.6)	M21(2.6)	M32(2.9)	UF13(2.9)	M41(3.2)	M51(3.2)	M45(3.2)
M56	ESSFmv2(2.3)	ESSFwk2(2.6)	SBSwk2(2.9)	ESSFwc1(2.9)	ESSFmm1(3.2	ESSFdc1(3.2)	SA33(3.2)	SA12(3.6)	ESSFdk(3.6)	ESSFwc4(3.6
Upper Foothills										
UF11	UF11(1.4)	LF13(4.4)								
UF12	UF13(0.8)	UF12(1.2)	UF14(1.4)	LF21(1.7)	UF24(2)	M21(2)	LF14(2.3)	LF22(2.9)	DM23(3.2)	LF15(3.2)
UF13	UF13(0.8)	M21(1.2)	UF24(1.4)	UF14(1.7)	SA11(1.7)	UF12(2.3)	M32(2.6)	LF21(2.9)	UF25(3.2)	LF14(3.6)
UF14	UF14(0.5)	UF24(1.2)	UF13(1.2)	M21(1.2)	LF21(1.4)	LF22(2)	UF12(2)	DM23(2.3)	M32(2.6)	LF14(2.6)
UF15	LF23(0.8)	UF15(1)	UF14(1.4)	UF24(1.7)	UF25(1.7)	M32(2.3)	LF22(2.3)	FP11(2.3)	M41(2.6)	M53(2.6)
UF24	UF24(0.5)	UF14(1.2)	M21(1.2)	UF13(1.4)	SA11(1.7)	M32(2.3)	UF25(2.3)	SA12(2.6)	M41(2.9)	UF12(2.9)
UF25	UF24(0.6)	UF25(1)	M41(2)	UF14(2)	UF15(2)	M32(2.3)	SA11(2.3)	SA12(2.6)	M53(2.6)	
