# Supplementary material for: Strategies for Reforestation under Uncertain Future Climates: Guidelines for Alberta, Canada
Source: PLoS One. 2011 Aug 10;6(8):e22977. doi: 10.1371/journal.pone.0022977 (PMC3154268; doi:10.1371/journal.pone.0022977)
Supplement: Table S8 — Table of best matching seed sources for 2020s climate. The multivariate Mahalanobis climate distance is given in parenthesis. (PDF) [file pone.0022977.s012.pdf]

Table S8. Table of best matching seed sources for 2020s climate. The multivariate Mahalanobis climate distance is given in parenthesis

Seed Zone	Choice 1	Choice 2	Choice 3	Choice 4	Choice 5	Choice 6	Choice 7	Choice 8	Choice 9	Choice 10
Northern Mixedwood										
NM11	NM11(2.5)	PAD11(3.9)	CM11(4.2)							
NM21	NM11(1.2)	PAD11(1.3)	CM11(1.5)	CM13(1.7)	LBH12(1.7)	AP11(1.9)	LBH11(2)	KU11(2.1)	NM21(2.6)	LBH21(2.8)
Central Mixedwood										
CM11	DM11(1.6)	CM12(2.1)	CM13(2.1)	PAD11(2.2)	CM11(2.3)	AP11(2.4)	CM21(3.3)	LBH11(4.3)		
CM12	DM11(1.5)	CM12(2)	CM21(2.3)	CM31(2.4)	LBH15(2.9)	CM24(3.1)	DM12(3.1)	LBH16(3.2)	CM22(3.8)	LBH14(4.1)
CM13	DM11(0.8)	CM13(1.8)	CM12(1.9)	CM21(2.5)	LBH16(2.7)	AP11(3.1)	PAD11(3.1)	CM11(3.2)	LBH15(3.4)	
CM21	CM31(1.3)	DM11(1.8)	LBH15(1.8)	CM24(1.9)	CM21(2.1)	CM12(2.4)	DM12(2.7)	CM33(2.9)	LBH16(3)	LF11(3)
CM22	CM24(0.6)	CM31(1.4)	LBH15(1.4)	CM21(1.7)	CM23(1.7)	LF11(1.8)	CM22(1.9)	DM12(2.1)	CM33(2.3)	DM11(2.3)
CM23	CM24(0.7)	CM23(1.5)	LF11(1.7)	CM31(1.8)	DM12(1.8)	LBH15(1.9)	CM22(2.2)	CM33(2.2)	LBH16(2.5)	
CM24	CM33(0.8)	CM31(1)	LF11(1.2)	CM24(1.5)	DM13(1.6)	CM32(1.7)	DM21(2)	LBH15(2.1)	DM12(2.3)	PRP11(2.6)
CM31	DM21(0.5)	CM32(0.6)	CP11(0.9)	DM22(1.1)	CM31(1.2)	CM33(1.2)	CM34(1.7)	CP12(1.8)	DM13(1.8)	PRP11(2)
CM32	DM22(0.6)	CM32(1)	CP11(1.4)	CM34(1.9)	CM35(2)	DM21(2)	LF15(2.6)	CM33(2.8)	CP12(2.8)	LF14(2.8)
CM33	CM32(0.6)	CM34(0.7)	DM22(0.9)	CM33(1)	CP11(1)	DM21(1)	CP12(1.6)	DM13(1.6)	LF12(2)	CM31(2.1)
CM34	CM34(0.5)	DM22(1.1)	CP11(1.4)	CM32(1.6)	CP12(1.7)	LF14(1.8)	DM21(2.2)	DM23(2.2)	LF12(2.2)	LF15(2.2)
CM35	LF13(1)	CM35(1.3)	LF15(2.1)	LF14(2.7)	DM22(3.4)	UF12(3.5)	LF21(4.4)			
Dry Mixedwood										
DM11	DM11(1.7)	CM31(2.2)	DM12(2.3)	CM12(2.9)	LBH16(2.9)	LBH15(3)	CM21(3.1)	DM21(3.4)	CM24(3.6)	PRP11(3.7)
DM12	PRP11(0.4)	DM13(0.6)	DM12(1)	DM21(1.3)	CM33(1.8)	CM31(1.9)	CP12(1.9)	NF11(2)	LF12(2.1)	CP11(2.3)
DM13	DM13(0.6)	CM34(0.9)	PRP11(0.9)	DM21(1)	CP12(1.1)	CM33(1.2)	CP11(1.2)	LF12(1.7)	CM32(1.8)	NF11(2)
DM21	CP11(0.7)	DM22(0.8)	DM21(1.1)	CM32(1.5)	CP12(1.6)	NF11(2.1)	CM34(2.3)	LF12(2.6)	CM33(3)	CM35(3.1)
DM22	DM22(1.1)	CM35(1.5)	CP11(2.1)	LF13(2.1)	LF15(2.4)	CM32(2.6)	LF14(2.7)	DM21(3.5)	CM34(3.6)	CP12(3.7)
DM23	CM35(0.3)	LF15(0.4)	LF14(0.8)	DM23(1.1)	LF13(1.3)	LF21(1.4)	LF22(1.8)	UF12(1.8)	DM22(2)	UF14(2.4)
Boreal Highlands										
BSA11	LBH12(1.1)	NM11(1.8)	KU11(2.5)	LBH21(3.2)	PAD11(3.2)	CM11(3.3)	AP11(3.7)	CM13(3.8)	LBH11(3.8)	NM21(4.2)
BSA12	NM11(0.7)	LBH12(1.2)	KU11(1.6)	NM21(2)	LBH21(2.1)	PAD11(3.1)	CM11(3.3)	BSA12(3.7)	LBH11(3.7)	CM13(3.8)
LBH11	DM11(0.4)	CM13(0.7)	CM12(1.1)	CM21(1.6)	AP11(1.8)	CM11(1.9)	LBH11(2)	PAD11(2)	UBH12(2)	LBH16(2.4)
LBH12	NM11(1.8)	PAD11(2.3)	CM11(2.5)	CM13(2.5)	LBH12(2.8)	AP11(2.9)	KU11(3.2)	LBH11(3.7)		
LBH13	CM21(0.4)	CM22(0.5)	LBH14(0.7)	CM23(0.8)	CM24(0.8)	CM12(1.1)	LBH15(1.1)	DM11(1.2)	UBH12(1.2)	LBH16(1.5)
LBH14	DM12(0.7)	LBH16(0.7)	LBH15(1.1)	CM24(1.3)	CM31(1.3)	DM11(1.6)	LBH14(1.7)	CM21(1.9)	CM23(2.1)	CM22(2.3)
LBH15	CM31(0.2)	CM33(0.6)	DM21(0.6)	CM32(0.7)	DM13(1.3)	LBH15(1.5)	CP11(1.6)	LF11(1.7)	PRP11(1.7)	DM12(1.9)
LBH16	DM12(0.6)	PRP11(0.8)	DM13(1.2)	LBH16(1.3)	UBH13(1.3)	CM31(1.5)	DM21(1.8)	CM33(2.1)	LF12(2.1)	LBH15(2.6)
LBH21	NM11(1.4)	LBH12(1.5)	CM13(1.7)	PAD11(1.7)	CM11(1.8)	LBH11(1.9)	AP11(2.2)	KU11(2.4)	LBH21(2.7)	NM21(2.8)
UBH11	UBH12(0.4)	CM21(0.7)	LBH14(0.8)	CM12(1)	DM11(1.1)	LBH16(1.2)	CM22(1.4)	LBH15(1.6)	CM13(1.9)	CM23(2.1)
UBH12	LBH16(0.5)	LBH15(1.1)	DM11(1.2)	DM12(1.3)	UBH13(1.5)	CM21(1.6)	CM31(1.6)	LBH14(1.7)	UBH12(1.7)	CM12(1.9)
UBH13	LF12(0.9)	UBH13(1.1)	PRP11(1.5)	DM21(2)	DM12(2.4)	DM13(2.4)	CP12(2.5)	NF11(2.5)	CM31(2.6)	CP11(2.6)
Lower Foothills										
LF11	CM33(0.4)	CM32(0.6)	CM31(1.4)	LF11(1.4)	DM21(1.5)	DM13(1.6)	DM22(1.8)	CP11(2)	PRP11(2.6)	CP12(2.7)
LF12	LF12(0.7)	CP11(1)	CP12(1.1)	CM34(1.4)	DM22(1.5)	DM21(1.8)	NF11(1.8)	PRP11(2.3)	LF14(2.4)	CM32(2.7)
LF13	LF13(0.8)	CM35(2.5)	UF12(2.9)	LF14(3.2)	LF15(3.2)					
LF14	LF14(0.5)	LF13(0.7)	CM35(0.9)	LF15(0.9)	UF12(1)	LF21(1.8)	DM23(2.3)	DM22(2.6)	UF13(2.7)	LF22(2.9)
LF15	CM35(0.7)	LF13(0.7)	LF15(1.1)	LF14(1.6)	UF12(2.1)	LF21(2.6)	DM22(3.1)	DM23(3.2)	LF22(3.5)	UF14(3.9)
LF21	LF15(0.5)	LF14(0.7)	LF21(0.8)	UF12(0.8)	CM35(0.9)	LF13(1.1)	LF22(1.6)	DM23(1.8)	UF14(1.8)	UF13(2.4)
LF22	LF15(0.3)	CM35(0.6)	LF21(0.8)	LF14(1)	LF22(1)	DM23(1.3)	LF13(1.8)	UF12(1.8)	UF14(1.9)	DM22(2.9)
LF23	LF22(0.6)	LF23(0.9)	DM23(1.2)	LF21(1.2)	UF14(1.5)	UF15(1.5)	FP11(2.1)	M32(2.4)	LF15(2.6)	LF14(2.9)
Montane										
M11	M11(0.7)	MG13(2.3)								
M21	M21(0.5)	UF13(1)	UF12(2.5)	UF14(2.5)	UF24(3.3)	LF14(3.7)	LF13(4.2)	SA11(4.2)	LF21(4.4)	
M22	M22(1.1)	FF11(1.2)	MG11(1.2)	LF12(1.5)	M32(1.5)	CP12(1.6)	M55(1.6)	M45(1.7)	NF11(2)	MG12(2.2)
M32	M32(0.9)	CP11(3)	DM23(1.3)	LF14(1.3)	UF14(1.4)	M22(1.6)	M45(1.6)	UF13(1.6)	LF12(1.7)	LF21(1.7)
M41	M32(0.5)	M45(0.7)	M55(0.8)	M41(1)	M22(1.2)	UF24(1.2)	M51(1.5)	FP12(1.6)	FF11(1.8)	
M42	UF24(1.4)	UF14(1.8)	FP12(2.2)	UF25(2.5)	M42(2.8)	SA12(2.9)	M32(3.2)	M41(3.2)	LF21(3.5)	LF22(3.5)
M43	FP11(0.5)	M44(0.5)	M43(0.7)	FF11(1)	M54(1)	UF15(1)	M32(1.1)	M45(1.1)	M53(1.1)	LF23(1.3)
M44	FP11(0.8)	M44(0.9)	FF11(1.1)	M45(1.1)	M32(1.2)	UF15(1.3)	FP12(1.4)	ICHmk3(1.4	M43(1.4)	M54(1.4)
M45	M56(1)	FP12(1.1)	M45(1.2)	M55(1.2)						
M51	M45(0.7)	M55(0.8)	FP12(1)	M32(1)	M56(1.1)	M51(1.2)	M41(1.3)	UF24(1.8)	M22(1.9)	
M53	M45(0.7)	M32(0.8)	FP12(0.9)	M55(1.2)	M54(1.3)	M53(1.4)	M51(1.5)	FF11(1.6)	M41(1.6)	M44(1.6)
M54	M45(0.4)	FP12(0.8)	M55(0.8)	M32(1)	FF11(1.4)	M54(1.4)	M51(1.5)	M56(1.5)	M41(1.6)	M53(1.7)
M55	M56(0.9)	M55(1.1)	FP12(1.6)	M45(1.6)	SA33(2.3)	M32(2.7)	FF11(3)	LF12(3.1)	M51(3.1)	M22(3.3)
M56	M56(1.2)	FP12(1.9)	ICHmk1(1.9)	MSdk(1.9)	SA33(2)	SBSwk2(2.1)	ICHwk1(2.2)	17x(2.3)	ESSFwc4(2.3)	SBSvk(2.4)
Upper Foothills										
UF11	UF11(1.1)									
UF12	LF13(0.7)	UF12(0.9)	LF14(1.9)	CM35(2.2)	LF15(2.2)	UF13(2.3)	LF21(3.1)	UF14(3.4)	M21(4.3)	
UF13	UF13(0.7)	UF12(0.8)	LF13(1.6)	M21(1.8)	LF14(2)	UF14(2.4)	LF15(3)	CM35(3.1)	LF21(3.2)	UF24(3.9)
UF14	UF12(0.8)	UF14(1.1)	LF13(1.3)	LF14(1.3)	UF13(1.3)	LF15(1.4)	CM35(1.5)	LF21(1.7)	DM23(2.4)	M21(2.4)
UF15	LF22(0.7)	LF23(0.9)	UF15(1.1)	DM23(1.2)	LF21(1.2)	UF14(1.2)	M32(1.5)	FP11(1.7)	M44(2.2)	UF24(2.2)
UF24	UF13(0.6)	UF14(0.9)	UF12(1)	M21(1.3)	LF14(1.6)	UF24(1.8)	LF13(2)	LF15(2.3)	LF21(2.3)	CM35(2.4)
UF25	UF24(0.6)	UF14(0.7)	M32(1.1)	UF25(1.5)	LF21(1.7)	UF13(1.7)	FP12(1.8)	LF22(1.9)	DM23(2.1)	M41(2.1)
