# Supplementary material for: Strategies for Reforestation under Uncertain Future Climates: Guidelines for Alberta, Canada
Source: PLoS One. 2011 Aug 10;6(8):e22977. doi: 10.1371/journal.pone.0022977 (PMC3154268; doi:10.1371/journal.pone.0022977)
Supplement: Table S9 — Table of best matching seed sources for 2050s climate. The multivariate Mahalanobis climate distance is given in parenthesis. (PDF) [file pone.0022977.s013.pdf]

Table S9. Table of best matching seed sources for 2050s climate. The multivariate Mahalanobis climate distance is given in parenthesis.

Seed Zone	Choice 1	Choice 2	Choice 3	Choice 4	Choice 5	Choice 6	Choice 7	Choice 8	Choice 9	Choice 10
Northern Mixedwood										
NM11	BWBSmw2(3.8)									
NM21	CM13(2.1)	PAD11(2.5)	CM11(2.7)	AP11(3.1)	DM11(3.1)	LBH11(3.8)	CM12(4.3)	NM11(4.3)		
Central Mixedwood										
CM11	DM11(3.9)	CM31(4.8)								
CM12	CM31(3)	DM21(3.3)	DM12(4.1)	CM32(4.3)	CM33(4.5)	PRP11(4.6)	DM13(4.7)	DM11(4.9)		
CM13	CM31(3)	DM11(3)	DM12(3.2)	DM21(3.4)	LBH16(3.8)	PRP11(4.1)	LBH15(4.4)	CM12(4.5)	UBH13(4.6)	DM13(4.8)
CM21	CM31(2.2)	DM21(2.5)	CM32(2.8)	CM33(3.1)	DM13(3.8)	DM12(3.9)	CP11(4)	CM24(4.1)	DM22(4.1)	LBH15(4.1)
CM22	CM31(1.6)	CM33(1.9)	CM32(2.2)	DM21(2.3)	DM13(2.5)	CM24(2.6)	LF11(2.8)	DM12(2.9)	LBH15(3.2)	PRP11(3.3)
CM23	CM33(1.8)	CM31(2)	DM13(2)	DM21(2.3)	CM32(2.6)	DM12(2.6)	PRP11(2.6)	LF11(2.9)	CM24(3)	
CM24	CM32(1.5)	CM33(1.7)	DM21(1.9)	DM13(2.2)	CM31(2.4)	DM22(2.4)	CM34(2.5)	CP11(2.5)	PRP11(3.1)	LF11(3.3)
CM31	DM22(1.2)	CP11(1.4)	DM21(1.8)	CM32(1.9)	CP12(2.6)	CM34(2.8)	NF11(3.2)	CM33(3.3)	CM35(3.6)	DM13(3.6)
CM32	DM22(1.8)	CM35(2.5)	CM32(3)	CP11(3)	LF13(3.3)	LF15(3.4)	CM34(3.9)	LF14(3.9)	DM21(4.2)	CP12(4.5)
CM33	DM22(1)	CP11(1.6)	CM32(1.7)	CM34(1.8)	DM21(2.3)	CP12(2.5)	CM35(2.6)	CM33(3)	LF14(3.1)	LF15(3.1)
CM34	DM22(1.6)	CM35(1.8)	CM34(2)	LF15(2.1)	LF14(2.2)	CP11(2.4)	CM32(2.9)	DM23(2.9)	CP12(3)	LF13(3)
CM35	CM35(3.8)	LF13(3.3)								
Dry Mixedwood										
DM11	DM21(2.6)	CM31(2.9)	DM12(3.4)	PRP11(3.5)	DM13(3.9)	CP11(4.1)	CM32(4.2)	CM33(4.3)	NF11(4.9)	
DM12	PRP11(1.2)	DM21(1.4)	CP11(1.5)	DM13(1.5)	CP12(1.6)	NF11(1.7)	LF12(2.2)	CM34(2.5)	DM22(2.6)	CM33(2.7)
DM13	CP11(1.3)	CP12(1.5)	DM22(1.5)	CM34(1.6)	DM21(1.9)	DM13(2.2)	CM32(2.4)	PRP11(2.4)	NF11(2.5)	LF12(2.7)
DM21	DM22(2)	CP11(2.3)	CM35(3.4)	CP12(3.4)	DM21(3.4)	NF11(3.7)	CM32(3.8)	LF13(4.2)	CM34(4.5)	LF15(4.7)
DM22	CM35(2.9)	CP11(4.6)	DM22(3.3)	LF13(3.2)	LF14(4.8)	LF15(4.2)				
DM23	CM35(1.3)	LF13(1.7)	LF15(1.9)	LF14(2.7)	DM23(3.3)	LF21(3.5)	UF12(3.7)	DM22(3.8)	LF22(4)	UF14(4.7)
Boreal Highlands										
BSA11	BWBSdk2(2.5)	BWBSmw2(3)	NM11(4.7)	CM13(4.8)						
BSA12	NM11(2.8)	CM13(3.6)	PAD11(3.7)	CM11(4)	LBH12(4.3)	AP11(4.6)	KU11(4.9)			
LBH11	DM11(1.6)	LBH16(2.4)	DM12(2.6)	CM12(2.8)	CM31(2.8)	CM21(3.2)	LBH15(3.3)	CM13(3.6)	UBH13(3.6)	
LBH12	BWBSmw2(2.5	BWBSdk2(3.3)								
LBH13	CM31(0.9)	DM12(1.5)	CM24(1.6)	LBH15(1.7)	CM33(1.8)	LBH16(2.1)	LF11(2.1)	DM13(2.3)	DM21(2.3)	CM32(2.7)
LBH14	DM12(1.2)	CM31(1.3)	PRP11(1.3)	DM13(1.4)	DM21(1.4)	CM33(1.9)	LBH16(2.5)	CM32(2.7)	CP11(2.7)	LBH15(2.9)
LBH15	DM21(0.7)	CM32(0.8)	DM22(0.9)	CP11(1)	CM33(1.4)	CM31(1.6)	CM34(1.8)	DM13(1.9)	CP12(2)	PRP11(2.1)
LBH16	PRP11(0.9)	DM21(1.2)	DM13(1.5)	LF12(1.6)	CP11(1.8)	CP12(2)	DM12(2)	NF11(2)	CM31(2.5)	CM33(2.5)
LBH21	CM13(2.4)	PAD11(3.3)	CM11(3.5)	DM11(3.6)	AP11(3.8)	LBH11(4.2)	NM11(4.7)			
UBH11	CM31(1.1)	LBH16(1.1)	LBH15(1.4)	DM12(1.6)	UBH13(1.8)	DM11(2)	CM24(2.4)	CM21(2.5)	DM21(2.6)	CM33(2.7)
UBH12	CM31(1.4)	DM12(1.4)	DM21(1.7)	LBH16(1.9)	PRP11(1.9)	UBH13(1.9)	CP12(4)	DM13(2.3)	CM33(2.6)	LBH15(2.6)
UBH13	LF12(1.2)	NF11(2)	CP11(2.1)	CP12(2.1)	PRP11(2.1)	DM21(2.2)	DM13(3.2)	DM22(3.2)	CM34(3.4)	UBH13(3.4)
Lower Foothills										
LF11	CM32(1.1)	CM34(1.3)	DM22(1.3)	CM33(1.7)	CP11(2.1)	DM21(2.2)	DM13(2.8)	CM35(3.1)	CP12(3.1)	CM31(3.2)
LF12	CP11(1.5)	DM22(1.6)	CP12(1.7)	LF12(2)	CM34(2.2)	LF14(2.3)	NF11(2.4)	CM35(2.7)	DM21(2.8)	LF13(3)
LF13	LF13(3.1)	CM35(4.8)								
LF14	LF13(1.2)	CM35(1.6)	LF15(2)	LF14(2.1)	UF12(2.8)	LF21(3.5)	DM23(3.8)	DM22(4.1)	LF22(4.4)	UF13(4.9)
LF15	LF13(2.6)	CM35(3)	LF15(3.9)	LF14(4.8)						
LF21	LF13(1.4)	CM35(1.8)	LF15(1.9)	LF14(2.4)	UF12(2.5)	LF21(2.9)	LF22(3.8)	DM23(3.9)	UF14(4)	UF13(4.5)
LF22	CM35(1.5)	LF15(1.7)	LF13(1.8)	LF14(2.6)	LF21(3)	UF12(3.2)	LF22(3.4)	DM23(3.5)	UF14(4.1)	DM22(4.4)
LF23	LF22(0.8)	DM23(1)	LF15(1)	LF21(1)	CM35(1.6)	LF14(1.6)	UF14(1.6)	UF12(2.5)	LF23(2.7)	LF13(3)
Montane										
M11	43x(2)	M11(2.2)	17aj(2.3)	17al(2.3)	80b(2.5)	80c(2.5)	43w(2.6)	17ab(2.7)	18d(2.8)	18b(2.9)
M21	M21(1.8)	UF13(2.3)	UF12(3.1)	LF13(3.2)	UF14(4.3)	LF14(4.4)	UF11(4.4)			
M22	FF11(1.6)	MG11(1.6)	LF14(1.8)	CP12(1.9)	LF12(1.9)	M22(1.9)	M45(1.9)	DM23(2.1)	M32(2.1)	M55(2.2)
M32	LF14(0.8)	UF12(1.4)	UF13(1.4)	CM35(1.6)	DM23(1.6)	LF15(1.6)	LF13(1.7)	UF14(1.7)	LF21(1.8)	FP12(1.9)
M41	FP12(1.8)	M55(1.8)	UF13(1.8)	LF14(1.9)	M45(1.9)	M56(2)	M32(2.1)	UF14(2.1)	M21(2.3)	UF24(2.3)
M42	UF14(1.7)	LF14(1.8)	FP12(1.9)	CM35(2.1)	LF15(2.2)	UF12(2.2)	LF13(2.3)	LF21(2.4)	UF13(2.5)	UF24(2.7)
M43	FF11(1)	FP12(1)	DM23(1.3)	M45(1.3)	M32(1.5)	FP11(1.7)	LF22(1.9)	M22(2)	M44(2)	
M44	FP12(1)	DM23(1.4)	FF11(1.5)	M45(1.7)	LF22(1.9)	M32(1.9)	FP11(2.1)	LF21(2.1)	UF14(2.1)	
M45	FP12(1.6)	M56(2.1)	M45(2.6)	M55(2.8)	LF14(3.2)	FF11(3.4)	CM35(3.7)	LF12(3.7)	MG11(3.8)	CP12(3.9)
M51	M56(1.1)	FP12(1.4)	M55(1.7)	M45(1.9)	LF14(2.5)	LF12(2.6)	M32(2.6)	SA33(2.7)	UF13(2.7)	
M53	FP12(0.9)	M45(1.7)	LF14(1.8)	M56(1.8)	DM23(1.9)	M55(2)	M32(2.1)	UF14(2.2)	FF11(2.3)	
M54	FP12(0.7)	M45(1.5)	M56(1.7)	M55(1.9)	LF14(2.2)	M32(2.2)	DM23(2.3)	FF11(2.3)	UF14(2.3)	CM35(2.7)
M55	FP12(1.9)	M56(2.2)	M45(3)	M55(3)	UF13(3.5)	CM35(3.8)	LF12(3.9)	LF13(3.9)	SA33(3.9)	UF14(3.9)
M56	ICHmw2(1.9)	ESSFwc1(2.1)	ESSFwk2(2.8)	ICHdw(2.9)	SBSwk2(3)	15o(3.2)	BWBSwk1(3.2	ICHwk1(3.2)	17x(3.3)	80c(3.3)
Upper Foothills										
UF11	UF11(3.8)									
UF12	LF13(2.3)	UF12(3.7)	UF11(4.2)	CM35(4.4)	LF15(4.8)	LF14(4.9)				
UF13	LF13(1.8)	UF12(1.9)	UF13(2.3)	LF14(3.1)	M21(3.2)	CM35(3.6)	LF15(3.8)	UF14(3.8)	UF11(4.2)	LF21(4.4)
UF14	LF13(1.4)	UF12(2.1)	CM35(2.4)	LF15(2.7)	LF14(2.8)	UF13(2.9)	UF14(3)	LF21(3.6)	M21(4)	DM23(4.5)
UF15	LF21(0.7)	LF22(0.7)	DM23(0.8)	LF15(0.9)	UF14(1)	LF14(1.1)	CM35(1.4)	UF12(1.9)	FP12(2.3)	LF23(2.5)
UF24	LF13(1.5)	UF13(1.8)	UF12(1.9)	M21(2.5)	UF14(2.7)	LF14(2.8)	CM35(2.9)	LF15(3.3)	UF24(4)	
UF25	UF14(0.9)	LF14(1.2)	UF12(1.2)	UF13(1.2)	LF13(1.7)	LF15(1.7)	LF21(1.7)	CM35(1.8)	UF24(1.8)	DM23(2.1)
