# Supplementary material for: Strategies for Reforestation under Uncertain Future Climates: Guidelines for Alberta, Canada
Source: PLoS One. 2011 Aug 10;6(8):e22977. doi: 10.1371/journal.pone.0022977 (PMC3154268; doi:10.1371/journal.pone.0022977)
Supplement: Table S10 — Table of best matching seed sources for 2080s climate. The multivariate Mahalanobis climate distance is given in parenthesis. (PDF) [file pone.0022977.s014.pdf]

Table S10. Table of best matching seed sources for 2080s climate. The multivariate Mahalanobis climate distance is given in parenthesis.

Seed Zone	Choice 1	Choice 2	Choice 3	Choice 4	Choice 5	Choice 6	Choice 7	Choice 8	Choice 9	Choice 10
<u>Northern Mixedwood</u>										
NM11	BWBSmw2(5.9)	BWBSdk2(7)	BWBSmw1(8.9)							
NM21	DM11(3.8)	CM13(4.8)	CM12(5.4)							
<u>Central Mixedwood</u>										
CM11	42i(5.2)									
CM12	42i(3.8)	42k(4.2)	DM21(4.3)	CM32(5.2)	CP11(5.2)	CM31(5.3)				
CM13	DM21(2.9)	CM31(3.7)	PRP11(3.8)	CP11(4)	DM12(4)	DM13(4.5)	NF11(4.5)	CM32(4.8)	DM22(4.9)	
CM21	DM21(3.7)	CM32(3.8)	DM22(4.2)	CP11(4.4)	CM31(4.6)	CM33(4.9)	DM13(5.2)			
CM22	CM32(2.8)	DM21(3)	CM33(3.4)	DM22(3.4)	CP11(3.6)	DM13(3.6)	CM31(3.7)	PRP11(4.3)	CM34(4.5)	CP12(5)
CM23	DM21(2.8)	CM32(2.9)	DM13(3.1)	CM33(3.2)	CP11(3.2)	DM22(3.3)	PRP11(3.7)	CM31(3.9)	CM34(3.9)	
CM24	DM22(2.6)	CM32(2.7)	CP11(3.1)	DM21(3.3)	CM34(3.5)	CM33(3.7)	DM13(3.9)	CP12(4.2)	PRP11(4.7)	
CM31	DM22(2.4)	CP11(2.8)	CP12(3.7)	DM21(3.7)	CM32(3.8)	CM35(3.9)	CM34(4.2)	NF11(4.3)	LF13(4.8)	LF15(5)
CM32	CM35(3.7)	DM22(3.7)	LF13(4.6)	LF15(4.6)	CP11(5)	CM32(5.2)				
CM33	DM22(2.2)	CM35(2.8)	CP11(3.2)	CM32(3.5)	LF15(3.5)	CM34(3.6)	LF13(3.8)	LF14(4)	CP12(4.1)	DM21(4.4)
CM34	DM22(3)	CM35(2.5)	DM23(4)	LF15(3)	LF14(3.5)	CM34(3.7)	LF13(3.7)	CP11(4.1)	CP12(4.6)	LF21(4.6)
CM35	17b(3.6)	17a(5.1)								
<u>Dry Mixedwood</u>										
DM11	42i(2.8)	42k(2.9)	DM21(3.4)	CP11(4)	DM22(4.6)	NF11(4.6)	PRP11(4.6)	CM32(4.8)	CM31(4.9)	DM13(4.9)
DM12	CP11(1.7)	CP12(1.9)	NF11(2)	DM22(2.3)	DM21(2.4)	PRP11(2.7)	DMG11(2.9)	DM13(3)	CM34(3.1)	LF12(3.3)
DM13	DM22(2.2)	CP11(2.3)	CP12(2.7)	CM34(2.9)	DM21(3.6)	CM32(3.7)	CM35(3.7)	NF11(3.7)	DM13(4.1)	MG11(4.3)
DM21	DM22(3.7)	CP11(4)	CM35(4.4)	CP12(4.8)	LF13(4.9)	NF11(5.1)				
DM22	CM35(4.3)	LF13(4.5)	DM22(5.2)							
DM23	CM35(3)	LF13(3.3)	LF15(3.7)	LF14(4.7)	DM23(5.2)	LF21(5.3)				
<u>Boreal Highlands</u>										
BSA11	BWBSdk2(2.7)	BWBSmw2(2.8)								
BSA12	BWBSmw2(2.2)	BWBSdk2(3.8)	CM13(5.1)							
LBH11	DM21(2.6)	DM12(2.8)	CM31(2.9)	PRP11(3.3)	DM13(3.9)	LBH16(4)	CP11(4.1)	CM32(4.6)	CM33(4.6)	UBH13(4.7)
LBH12	BWBSmw2(3.6)	BWBSdk2(4.6)								
LBH13	DM21(1.7)	CM31(1.9)	CM32(2)	CM33(2)	DM13(2.3)	PRP11(2.6)	CP11(2.7)	DM22(2.8)	DM12(2.9)	LF11(3.4)
LBH14	DM21(1.5)	CP11(1.9)	PRP11(2)	DM13(2.2)	DM22(2.5)	CM32(2.7)	CP12(2.7)	NF11(2.7)	CM31(2.9)	CM33(2.9)
LBH15	DM22(1.3)	CP11(1.5)	CM32(2)	DM21(2)	CP12(2.5)	CM34(2.7)	CM35(3.1)	CM33(3.2)	NF11(3.3)	DM13(3.4)
LBH16	CP11(1.5)	DM21(1.7)	CP12(1.8)	NF11(1.8)	PRP11(1.9)	LF12(2.2)	DM22(2.3)	DM13(2.4)	CM34(2.9)	DMG11(3.1)
LBH21	BWBSmw2(2.9)	BWBSdk2(4.3)	DM11(4.5)	LBH16(5.2)	UBH13(5.2)	CM13(5.3)				
UBH11	DM21(1.2)	CM31(1.4)	PRP11(2)	CM32(2.1)	DM12(2.1)	CM33(2.2)	CP11(2.2)	DM13(2.2)	DM22(2.6)	LF12(2.8)
UBH12	DM21(1.5)	CP11(1.9)	PRP11(2.2)	DM22(2.6)	LF12(2.7)	NF11(2.7)	CM31(2.8)	DM13(2.8)	CP12(2.9)	CM32(3)
UBH13	LF12(1.8)	CP11(2)	NF11(2)	CP12(2.1)	DM22(2.8)	DM21(2.9)	PRP11(3.2)	CM34(3.5)	MG11(3.6)	DMG11(3.7)
<u>Lower Foothills</u>										
LF11	DM22(2)	CM32(2.6)	CM34(2.7)	CM35(2.9)	CP11(3.1)	LF15(3.3)	LF14(3.7)	CM33(3.9)	LF13(3.9)	DM21(4)
LF12	DM22(2.2)	CM35(2.4)	CP11(2.5)	CP12(2.7)	LF13(2.7)	LF14(2.7)	LF15(3)	CM34(3.2)	DM23(3.5)	LF12(3.6)
LF13	17b(5.9)	LF13(6.1)	CM35(7.2)	UF11(7.2)	LF15(8)					
LF14	LF13(2.9)	CM35(3)	LF15(3.4)	LF14(3.9)	LF21(4.8)	UF12(4.8)	DM23(5.1)			
LF15	17b(4)	LF13(5)	CM35(5.4)							
LF21	LF13(3.3)	CM35(3.6)	LF15(3.9)	LF14(4.8)	UF12(5.1)	LF21(5.2)				
LF22	CM35(3.3)	LF13(3.6)	LF15(3.8)	LF14(4.9)	LF21(5.3)					
LF23	LF15(1.7)	CM35(1.8)	LF22(2.2)	DM23(2.3)	LF21(2.3)	LF14(2.7)	LF13(2.9)	UF14(3.1)	UF12(3.7)	
<u>Montane</u>										
M11	43w(1.7)	43x(2)	80c(2.7)	80b(2.9)	43o(3.1)	17a(3.2)	17aj(3.2)	18b(3.2)	17al(3.3)	43q(3.3)
M21	17t(1.9)	M21(3.4)	UF11(4)	UF13(4.3)	LF13(4.4)	UF12(4.9)				
M22	IDFmw1(1.9)	IDFmw2(2)	15c(2.2)	PPdh2(2.2)	42q(2.3)	BWBSwk1(2.3)	IDFdm2(2.4)	MG11(2.4)	LF14(2.5)	CM35(2.6)
M32	LF13(1.3)	LF14(1.6)	CM35(1.7)	LF15(2.1)	UF12(2.2)	DM23(2.6)	UF13(2.6)	FP12(2.7)	LF21(2.9)	UF14(2.9)
M41	BWBSwk1(0.9)	LF13(1.9)	UF13(2.3)	LF14(2.4)	CM35(2.6)	FP12(2.6)	UF12(2.6)	M21(2.7)	UF14(2.8)	
M42	LF13(2.2)	CM35(2.7)	LF14(3.1)	LF15(3.4)	UF12(3.4)	FP12(3.5)	UF14(3.5)	UF13(3.8)	LF21(4.4)	M56(4.5)
M43	FP12(1.5)	CM35(1.6)	DM23(1.6)	LF15(1.8)	LF14(1.9)	FF11(2.2)	LF21(2.3)	LF22(2.3)	M45(2.5)	UF14(2.7)
M44	FP12(1.7)	CM35(1.8)	DM23(1.8)	LF15(1.9)	LF14(2.1)	LF21(2.4)	LF22(2.4)	UF14(2.7)	FF11(2.8)	LF13(3)
M45	43v(2.1)	17a(2.4)	ICHmw2(2.6)	BWBSwk1(2.8)	FP12(2.8)	ICHdw(2.9)	15c(3)	43q(3.1)	ICHmk1(3.1)	IDFmw1(3.3)
M51	BWBSwk1(1.1)	SBSwk2(1.8)	ESSFmv2(1.9)	FP12(1.9)	ICHmw2(1.9)	ICHmk1(2.1)	M56(2.1)	ESSFwc1(2.2)	ICHmm(2.2)	MSdk(2.4)
M53	FP12(1.4)	CM35(1.7)	LF14(2)	LF15(2.1)	LF13(2.3)	DM23(2.5)	UF14(2.7)	M56(2.8)	LF21(2.9)	M45(2.9)
M54	FP12(1.7)	CM35(2.2)	LF14(2.5)	LF13(2.7)	LF15(2.7)	DM23(3)	M56(3)	M45(3.1)	UF14(3.2)	LF21(3.5)
M55	17a(2.1)	BWBSwk1(2.3)	43v(2.7)	17b(3.1)	17t(3.2)	ICHmw2(3.2)	ESSFmv2(3.3)	43q(3.4)	43d(3.5)	FP12(3.5)
M56	17a(3.6)	ICHmw2(3.6)	15o(3.9)	BWBSwk1(4.1)	ESSFwc1(4.1)	80c(4.2)	ICHdw(4.4)	ESSFmv2(4.5)	17b(4.6)	
<u>Upper Foothills</u>										
UF11	UF11(8.3)	17b(12.1)								
UF12	LF13(5.3)	UF11(5.4)								
UF13	LF13(3.2)	UF11(3.6)	UF12(4.2)	CM35(4.8)	UF13(4.8)	LF14(4.9)	LF15(5.1)	M21(5.2)		
UF14	LF13(3)	UF11(3.9)	CM35(4.1)	UF12(4.4)	LF15(4.6)	LF14(4.9)	UF14(5.1)	UF13(5.3)		
UF15	CM35(1.1)	LF15(1.2)	LF14(1.7)	LF21(1.7)	DM23(1.8)	LF13(1.8)	LF22(1.9)	UF14(2.1)	UF12(2.5)	FP12(3.1)
UF24	UF11(2.3)	LF13(2.5)	UF12(3.6)	UF13(3.7)	CM35(4.2)	M21(4.3)	LF14(4.5)	UF14(4.5)	LF15(4.8)	
UF25	LF13(1.4)	CM35(1.9)	LF14(2.1)	UF12(2.1)	LF15(2.3)	UF14(2.3)	UF13(2.5)	LF21(3)	DM23(3.3)	FP12(3.3)
