# Supplementary material for: Strategies for Reforestation under Uncertain Future Climates: Guidelines for Alberta, Canada
Source: PLoS One. 2011 Aug 10;6(8):e22977. doi: 10.1371/journal.pone.0022977 (PMC3154268; doi:10.1371/journal.pone.0022977)
Supplement: Table S11 — Locations of recommended seed choices which originate outside of Alberta. For British Columbia we report the relevant ecological “variants” and “zones” [18], and for the United States we report the corresponding state and “level III & IV” ecoregions [20]. (PDF) [file pone.0022977.s015.pdf]

Table S11. Locations of recommended seed choices which originate outside of Alberta. For British Columbia we report the relevant ecological "variants" and "zones" [18], and for the United States we report the corresponding state and "level III & IV" ecoregions [20].

Recommended seed choice	Zone/Ecoregion
British Columbia	
BWBSdk2, BWBSmw1, BWBSmw2, BWBSwk1	Boreal White and Black Spruce zone
ESSFmv2, ESSFwc1 ESSFwk2	Engelmann Spruce-Subalpine Fir zone
ICHdw, ICHmm, ICHmk1, ICHmw2, ICHwk1	Interior Cedar-Hemlock zone
IDFdm2, IDFmw1, IDFmw2	Interior Douglas-fir zone
MSdk	Montane Spruce zone
PPdh2	Ponderosa Pine zone
SBSwk2	Sub-Boreal Spruce zone
Idaho	
15o	Northern Rockies ecoregion
17ab	Middle Rockies ecoregion
80b, 80c	Northern Basin and Range ecoregion
Montana	
15c	Northern Rockies ecoregion
17al, 17aj, 17t, 17x	Middle Rockies ecoregion
42i, 42k, 42q	Northwestern Glaciated Plains ecoregion
43d, 43o, 43v	Northwestern Great Plains ecoregion
South Dakota	
17b	Middle Rockies ecoregion
Wyoming	
17a	Middle Rockies ecoregion
18b, 18d	Wyoming Basin ecoregion
43q, 43x, 43w	Northwestern Great Plains ecoregion
